# From chronic kidney disease classification to genuine early prediction: a systematic review of artificial intelligence, methodological quality, and translational evidence

**DOI:** 10.3389/frai.2026.1976937

**Published:** 2026-10-19

**Authors:** Aminu Bashir Suleiman, Ahmad Tijjani Garba, Aisha Mustapha Ahmad, Kaloma Usman Majikumna, Harisu Abdullahi Shehu, Huseyin Kusetogullari

**Affiliations:** 1 Department of Cyber Security, Federal University Dutsin-Ma, Katsina, Nigeria; 2 College of Intelligence and Computing, Tianjin University, Tianjin, China; 3 Department of Information Technology, Bayero University, Kano, Nigeria; 4 Department of Computer Engineering, King Mongkut's University of Technology Thonburi, Bangkok, Thailand; 5 Department of Computer Science, Bayero University, Kano, Nigeria; 6 School of Digital Engineering and Artificial Intelligence, Euromed University of Fes, Fes-Meknes, Morocco; 7 School of Engineering and Computer Science, Victoria University of Wellington, Wellington, New Zealand; 8 Department of Computer Science, Blekinge Institute of Technology, Karlskrona, Sweden

**Keywords:** chronic kidney disease, clinical prediction models, external validation, future-oriented prediction, prediction horizon

## Abstract

**Introduction:**

Artificial intelligence has been widely applied to chronic kidney disease (CKD), but the term ‘prediction” is frequently used for tasks ranging from contemporaneous disease classification to genuine forecasting of future renal outcomes. This systematic review examined this distinction while evaluating methodological quality and evidence for clinical translation.

**Methods:**

A predefined Scopus-based search covering 2021–2026 identified eligible AI- and machine-learning-based CKD studies. After screening and duplicate reconciliation, 169 unique primary studies were included. Future-oriented prediction required an identifiable baseline, predictors available at or before baseline, a subsequent renal outcome, and a meaningful prediction horizon. Studies were evaluated for modeling approaches, datasets, validation, calibration, explainability, clinical usefulness, and methodological quality using PROBAST+AI.

**Results:**

Of 169 studies, 122 (72.2%) addressed current-state CKD detection or classification, while only 46 (27.2%) contained a clearly future-oriented prediction component. Seventeen studies used geographic or institutional external validation and only four used an independent external cohort or dataset. Calibration was assessed in 12 studies and explicit explainability methods were reported in 16. PROBAST+AI identified high overall risk of bias in 156 studies during development and 164 during evaluation, with no study achieving an overall low-risk judgement.

**Discussion:**

CKD-AI evidence is substantially stronger for retrospective classification than for genuine early prediction or clinical translation. Future research should prioritize longitudinal prediction, independent and temporal validation, leakage-resistant analysis, calibration, clinically validated explainability, and prospective real-world evaluation.

## Highlights

- Genuine future prediction appeared in only 46 of 169 CKD-AI studies.
- Current-state classification dominated the CKD prediction literature.
- Public benchmark studies did not provide genuine future-oriented prediction.
- External validation and calibration were rarely reported.
- PROBAST+AI showed widespread high risk of bias.

## 1 Introduction

Chronic kidney disease (CKD) is a major global health problem that often progresses without obvious symptoms until substantial kidney damage has occurred. The Global Burden of Disease 2017 study estimated approximately 697.5 million CKD cases worldwide and 1.2 million associated deaths in 2017 (GBD Chronic Kidney Disease Collaboration, 2020). CKD is also closely associated with cardiovascular disease, diabetes, and hypertension, creating substantial long-term burdens for patients and healthcare systems. Because established kidney damage may be irreversible, identifying individuals at risk before substantial deterioration occurs is important for slowing progression and preventing complications [GBD Chronic Kidney Disease Collaboration, 2020; Kidney Disease: Improving Global Outcomes (KDIGO) CKD Work Group, 2024].

KDIGO defines CKD as abnormalities of kidney structure or function persisting for at least 3 months and having implications for health [Kidney Disease: Improving Global Outcomes (KDIGO) CKD Work Group, 2024]. Clinical classification considers the cause of disease, glomerular filtration rate (GFR) category, and albuminuria category, with routine assessment relying primarily on serum creatinine-derived estimated GFR (eGFR) and urine albumin-to-creatinine ratio (uACR) [Chen et al., 2019; Kidney Disease: Improving Global Outcomes (KDIGO) CKD Work Group, 2024]. Early identification remains challenging because patients may have few symptoms, renal-function measures can vary over time, and chronicity must generally be established before CKD is confirmed.

These considerations make the distinction between disease classification and genuine prediction particularly important. Conventional models such as logistic regression and Cox proportional-hazards regression have long been used for renal-risk estimation. The Kidney Failure Risk Equation, for example, estimates future kidney-failure risk among individuals with established CKD using age, sex, eGFR, and uACR (Tangri et al., 2011). However, estimating future progression is conceptually different from classifying disease that is already present. A model should not be regarded as providing genuine early prediction when the predictors and CKD label are measured at approximately the same clinical time. Meaningful future-oriented prediction requires an identifiable index time, predictors available at or before that time, and an outcome occurring subsequently over a defined or inferable prediction horizon.

Artificial intelligence (AI) and machine learning (ML) offer opportunities to model non-linear relationships, complex interactions, longitudinal measurements, imaging, biomarkers, and other heterogeneous clinical information. Random forests, support-vector machines, boosting methods, neural networks, and hybrid approaches have consequently been applied extensively to CKD detection and prediction, while deep-learning methods have expanded CKD modeling to imaging, longitudinal electronic health records, and multimodal data. Nevertheless, greater algorithmic complexity does not necessarily translate into stronger clinical prediction. Comparative studies have shown that improvements over conventional regression may be modest and dependent on the population, outcome, and evaluation design (Cao et al., 2022; Chiu et al., 2021).

The interpretation of model performance is further complicated by differences in dataset provenance and validation. Near-perfect accuracy or AUROC can be achieved when models are repeatedly developed on small public benchmark datasets, whereas more heterogeneous longitudinal and externally validated cohorts often yield lower but more clinically informative estimates. Reliable prediction therefore requires more than discrimination alone; predictor timing, outcome definition, calibration, data leakage, validation independence, and clinical utility all influence whether reported performance is likely to generalize. Similarly, explainability methods such as SHAP and LIME can improve transparency but do not by themselves establish that model explanations are stable, clinically meaningful, or useful in decision-making (Ghosh and Khandoker, 2024).

Previous systematic reviews have examined CKD prediction and artificial intelligence from different perspectives, including model performance, validation, methodological quality, calibration, and clinical applicability (van Rijn et al., 2021; Schena et al., 2022; Sanmarchi et al., 2023; Pan and Tong, 2024). However, as summarized in Table 1, these reviews did not explicitly distinguish contemporaneous CKD classification from genuine future-oriented prediction using predefined temporal criteria. They also did not integrate predictor and outcome timing with a study-level audit of repeated dataset and benchmark reuse. Consequently, the temporal meaning and independence of the evidence underlying reported CKD “prediction” performance remain insufficiently synthesized.

**Table 1:** Comparison of the present review with previous reviews of CKD prediction and artificial intelligence.

Review characteristic	van Rijn et al. (2021)	Schena et al. (2022)	Sanmarchi et al. (2023)	Pan and Tong (2024)	Present review
Performance and validation	✓	✓	✓	✓	✓
Risk of bias	✓	✗	✓	✓	✓
Calibration and applicability	✓	✓	✓	✓	✓
Temporal distinction	✗	✗	✗	✗	✓
Dataset/temporal audit	✗	✗	✗	✗	✓

The present review addresses these gaps through a task-, temporality-, and methodology-oriented synthesis of 169 unique primary studies. In addition to examining performance, validation, risk of bias, calibration, and applicability, the review introduces an explicit temporal distinction between current-state classification and genuine future prediction and evaluates dataset dependence alongside predictor and outcome timing.

Accordingly, this systematic review aims to critically evaluate AI- and ML-based approaches for CKD detection and prediction, with particular emphasis on temporal validity, methodological quality, validation, and evidence for clinical translation.

To address this aim, the review is guided by the following research questions:

**RQ1:** Which machine-learning and deep-learning approaches have been applied to CKD detection and prediction, and how does their use vary across clinical tasks and data modalities?
**RQ2:** How should reported model performance be interpreted when differences in prediction task, outcome definition, dataset, evaluation metric, and validation design are considered?
**RQ3:** Which datasets, clinical predictors, preprocessing strategies, and feature-selection approaches characterize current AI-based CKD modeling?
**RQ4:** How are explainable-AI methods incorporated into CKD prediction models, and to what extent do they support clinical interpretation and decision-making?
**RQ5:** What methodological and translational limitations remain in AI-based CKD prediction, and which priorities are required to improve generalizability, transparency, and clinical implementation?

The main contributions of this review are as follows:

- It distinguishes contemporaneous CKD detection/classification from genuine future-oriented prediction using explicit temporal criteria.
- It interprets reported model performance in relation to prediction task, dataset provenance, validation design, calibration, and independence of evidence.
- It maps dataset reuse, predictor timing, preprocessing, feature selection, explainability, and potential information leakage across the evidence base.
- It evaluates methodological quality and applicability using PROBAST+AI across all 169 included studies.
- It synthesizes the evidence for generalizability and clinical translation rather than equating high benchmark performance with clinical usefulness.

## 2 Materials and methods

This systematic review examined artificial intelligence (AI) and machine-learning (ML) approaches for chronic kidney disease (CKD) detection, risk estimation, and outcome prediction. Particular attention was given to distinguishing current-state CKD classification from genuinely future-oriented prediction.

### 2.1 Review design and reporting framework

The review was conducted in accordance with PRISMA 2020, and search reporting followed PRISMA-S. CHARMS informed the extraction of prediction-model characteristics, while PROBAST+AI was used for methodological quality, risk-of-bias, and applicability assessment.

The review did not aim to identify a universally superior algorithm. Instead, models were interpreted according to clinical task, outcome definition, predictor timing, dataset provenance, validation design, and intended clinical use.

### 2.2 Review questions and PICOTS framework

The review was structured using the Population, Index model, Comparator, Outcome, Timing, and Setting (PICOTS) framework as shown in Table 2.

**Table 2:** PICOTS framework used in the review.

Element	Definition
Population	Human participants evaluated for CKD, renal impairment, incident CKD, CKD progression, kidney failure, dialysis, or related renal outcomes
Index model	AI or ML model used for CKD detection, classification, risk estimation, prognosis, or future renal-outcome prediction
Comparator	Alternative ML/DL methods, statistical models, clinical scores, clinician assessment, or no comparator
Outcome	CKD status/stage, incident CKD, renal-function decline, progression, ESKD/kidney failure, dialysis/RRT, or another clinically meaningful renal endpoint
Timing	Current-state assessment or prediction of a renal outcome occurring after a defined or inferable index point
Setting	Community, screening, hospital/EHR, registry, claims, imaging, laboratory, molecular, or multimodal clinical settings

### 2.3 Eligibility criteria

Primary studies were included when they used human-derived data, evaluated at least one AI or ML method, treated CKD or a clinically meaningful renal outcome as a substantive experimental target, and reported quantitative diagnostic or prognostic performance.

Reviews, meta-analyses, editorials, protocols, and other secondary publications were excluded. Studies were also excluded when CKD was only an incidental benchmark, when the modeled outcome was non-renal, when the study focused on population-level burden forecasting rather than individual prediction, or when the publication had been retracted.

Molecular and omics studies were retained only when molecular features were used to predict a defined patient-level renal outcome. Generic biomarker discovery, pathway analysis, immune-subtype studies, and kidney imaging studies without CKD-specific ground truth were excluded.

### 2.4 Information source and search strategy

Scopus was used as the sole bibliographic database for this review. The choice was based on the multidisciplinary nature of CKD-AI research, which spans nephrology, medicine, biomedical informatics, computer science, and engineering. Scopus provides broad cross-disciplinary coverage and indexes both journal and conference literature, making it suitable for retrieving studies across the clinical and computational domains relevant to this review.

Its use as the principal search source is also supported by previous evaluations of academic search systems. Gusenbauer and Haddaway (2020) systematically compared 28 search systems and identified Scopus among the databases suitable for evidence synthesis based on search functionality, reproducibility, and retrieval performance. A subsequent comparison of 56 bibliographic databases also demonstrated the broad disciplinary coverage of Scopus across major scientific fields (Gusenbauer, 2022). These characteristics were particularly relevant to the present review because restricting the search to a purely biomedical database could underrepresent computer-science and engineering studies on CKD prediction.

Nevertheless, reliance on a single database may have omitted eligible studies indexed exclusively elsewhere. Accordingly, the review was considered comprehensive within the predefined Scopus-based search strategy rather than exhaustive of all published CKD-AI literature. The search covered publications from 2021 to 2026 and combined three concept blocks using Boolean operators. The final Scopus search was executed on 3rd August, 2026. The complete search query was:


TITLE-ABS-KEY( ("chronic kidney disease"
OR CKD OR "chronic renal disease" OR
"chronic kidney insufficiency" OR "kidney
function decline" OR "renal impairment")
AND ("artificial intelligence" OR "machine
learning" OR "deep learning" OR "neural
network" OR CNN OR RNN OR LSTM OR GRU OR
transformer OR "decision tree" OR "random
forest" OR SVM OR "gradient boosting" OR
XGBoost OR LightGBM OR CatBoost OR KNN OR "Naive
Bayes" OR "logistic regression" OR ensemble OR
hybrid) AND ("early detection" OR screening
OR diagnosis OR prediction OR prognosis OR
classification OR "risk stratification" OR
progression OR staging OR "kidney failure" OR
"renal function decline" OR dialysis) ) AND
PUBYEAR > 2020 AND PUBYEAR < 2027


No additional bibliographic database was searched. The search results were subsequently screened according to the predefined eligibility criteria described in Section 2.3.

### 2.5 Study selection and eligibility reconciliation

Titles and abstracts were screened against the eligibility criteria, followed by full-text assessment where required. Publication identity was checked using title, authorship, DOI, and full-text content rather than filename alone.

Duplicate storage files were distinguished from duplicate publications. Duplicate publications were consolidated, whereas separate studies using the same underlying dataset were retained and coded for dataset dependence.

Two authors (ABS and ATG) independently screened records using predefined criteria. Uncertain records were retained for full-text review. Full-text exclusions were recorded with principal reasons. Disagreements were resolved via discussion, with a third author adjudicating unresolved conflicts (AMA).

### 2.6 Data extraction and coding

The extraction matrix recorded publication details, dataset, model, evaluation metrics, results, limitations, and key findings. Additional variables were extracted to address the research questions and reviewer concerns, including dataset provenance, prediction timing, validation design, calibration, explainability, clinical usefulness, deployment evidence, and dataset dependence (Table 3).

**Table 3:** Coding and data-extraction framework used in the review.

Coding domain	Extracted variables	Coding rule/purpose
Publication details	Author(s), year, title, publication source	Used to characterize the included evidence base and support study identification
Dataset characteristics	Dataset name, source, sample characteristics, dataset provenance	Used to distinguish public benchmarks, clinical cohorts, registries, population datasets, imaging datasets, and other data sources
Model characteristics	Machine-learning or deep-learning model, model family, comparator models	Used to classify the methodological approaches adopted across studies
Prediction task and timing	Clinical task, index time, predictor timing, outcome timing, prediction horizon	Used to distinguish current-state CKD classification from genuinely future-oriented prediction
Evaluation	Evaluation metrics, reported results, validation design	Used to interpret performance according to task, metric, and degree of validation independence
Calibration	Calibration assessment, calibration curve, Brier score, or related measures	Used to determine whether predicted risks were evaluated for agreement with observed outcomes
Explainability	XAI method, feature-attribution method, clinician interpretation or validation	Used to distinguish technical explainability from clinically validated interpretability
Clinical usefulness	Clinical comparator, decision-curve analysis, net benefit, utility assessment	Used to identify evidence that model performance translated into potential clinical value
Deployment evidence	Prototype, application, operational use, prospective or real-world evaluation	Used to assess the extent of translational and implementation evidence
Dataset dependence	Reuse of public benchmarks, common parent datasets, overlapping cohorts	Used to identify potentially non-independent evidence arising from repeated use of the same data source
Study findings	Main findings, reported strengths, limitations	Used to support qualitative synthesis of methodological strengths, weaknesses, and generalizability
Missing information	Not reported	Information not explicitly stated in the study was coded as “not reported” and was not inferred

A coding dictionary was used to standardize the extraction. Missing information was coded as “not reported” rather than inferred. The complete study-level data extraction, including study characteristics, datasets, modeling approaches, evaluation measures, temporal design, validation, calibration, explainability, clinical usefulness, deployment evidence, and dataset dependence, is provided in Supplementary Table S1.

### 2.7 Temporal classification of prediction tasks

Each study was classified according to the temporal relationship between predictors and renal outcomes.

A study was considered future-oriented only when an index time was defined or inferable, predictors were available at or before that index, the renal outcome occurred later, and a follow-up period or prediction horizon was identifiable.

The following categories were used in Table 4:

**Table 4:** Temporal classification of CKD prediction studies.

Code	Category	Definition
T1	Current-state detection	Predictors and CKD/renal label corresponded to approximately the same clinical time
T2	Incident CKD prediction	Participants were free of CKD at baseline and later developed CKD
T3	CKD progression/prognosis	Participants had CKD or renal disease at baseline and a later renal outcome was predicted
T4	Mixed design	The study combined current-state detection with a genuine future-oriented component
T5	Temporally ambiguous	Longitudinal information was used, but a clear baseline-to-future outcome structure was not established

Terms such as “prediction,” “forecasting,” “risk,” “real-time,” or the use of LSTM, GRU, RNN, or other temporal architectures were not considered sufficient evidence of future prediction.

To improve reproducibility, the temporal classification procedure was operationalized using the decision framework shown in Figure 1. Studies were classified according to the presence of an identifiable index time, predictor timing relative to the index time, outcome timing, and the availability of a follow-up period or prediction horizon.

**Figure 1:**
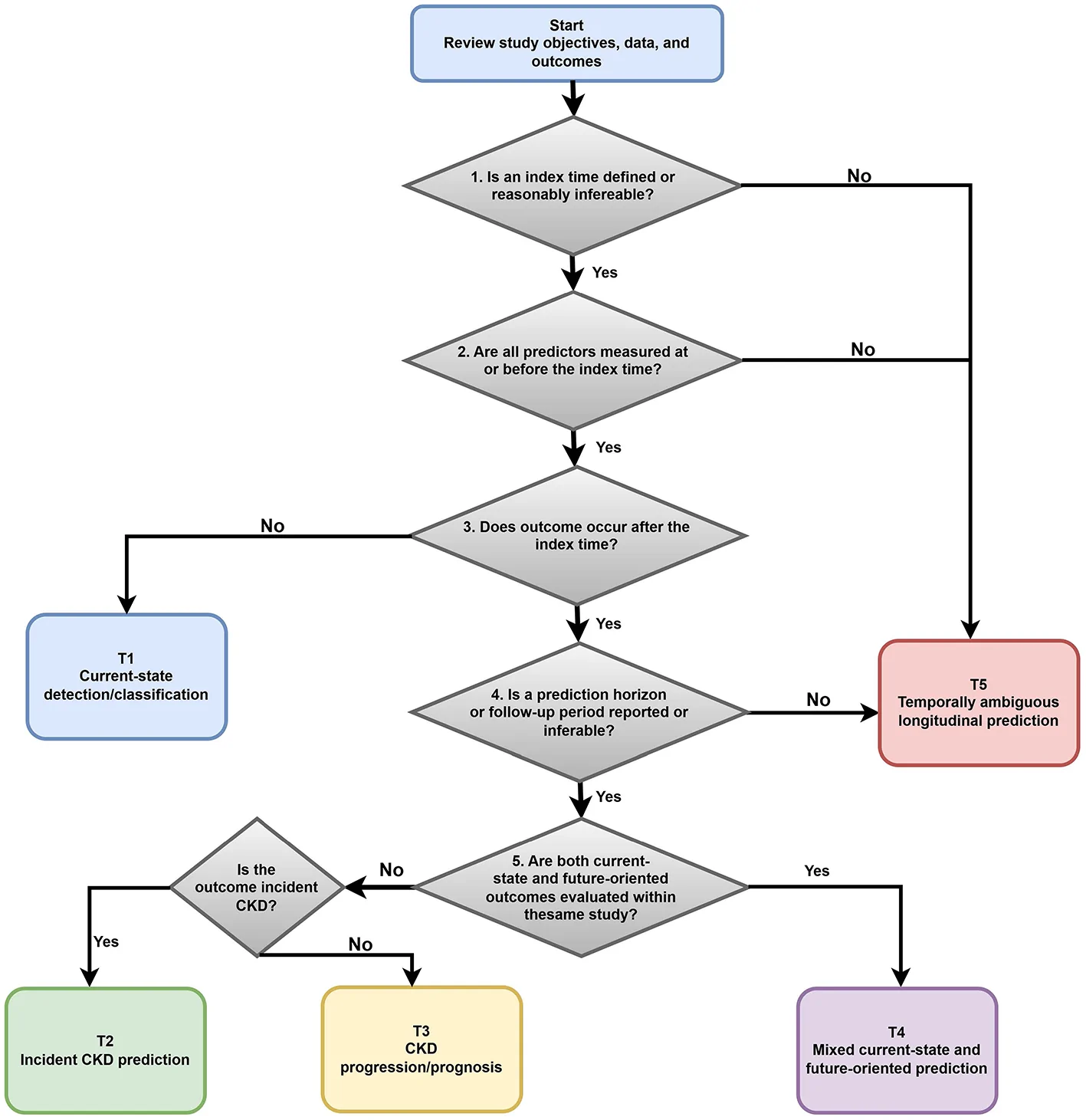
Decision tree for temporal classification of CKD prediction studies.

Using this framework, each included study was assigned to one of five mutually exclusive temporal classes (T1–T5), as defined in Table 4. The complete study-level temporal coding, including index time, predictor window, outcome timing, prediction horizon, and final T1–T5 classification for all 169 included studies, is provided in Supplementary Table S2.

### 2.8 Dataset provenance and dependency

Datasets were grouped as UCI CKD benchmark, other public benchmark, single-center clinical cohort, multicenter cohort, registry/EHR/claims source, population cohort, imaging dataset, molecular/omics dataset, or synthetic dataset.

Dataset dependence was coded separately to distinguish independent cohorts from repeated reuse of public benchmark data or the same clinical cohort. This prevented repeated evaluations on the same dataset from being interpreted as independent clinical replication.

### 2.9 Validation, explainability, and clinical translation

Validation was categorized as no clear validation, cross-validation, internal holdout, temporal validation, geographic/institutional external validation, or independent external-cohort validation.

Calibration, explainability, clinical usefulness, and deployment evidence were coded separately. Explainability included feature importance, SHAP, LIME, combined SHAP/LIME, and other interpretable methods. Clinical usefulness included comparison with established clinical scores, decision-curve analysis, clinician comparison, or implementation in a clinical tool or workflow.

### 2.10 Methodological quality and risk of bias

Methodological quality, risk of bias, and applicability were assessed using PROBAST+AI. The appraisal covered four domains: participants and data sources, predictors, outcome, and analysis. Development studies were assessed for methodological quality and applicability, while model-performance evaluations were assessed for risk of bias and applicability. Signaling questions were rated as “yes,” “probably yes,” “probably no,” “no,” “no information,” or “not applicable,” as appropriate.

Domain-level judgements were classified as low, high, or unclear concern for model development and as low, high, or unclear risk of bias for model evaluation. The assessments were independently reviewed by two reviewers, and disagreements were resolved through discussion and consensus. Judgements were retained at the domain level and were not converted into a numerical quality score. The complete study-level PROBAST+AI assessment, including domain-level judgements and signaling-question responses for all 169 included studies, is provided in Supplementary Table S3.

### 2.11 Data synthesis

The evidence was synthesized descriptively because the included studies differed substantially in population, clinical task, outcome, prediction horizon, dataset, validation design, and evaluation metric.

Counts and percentages were used for categorical characteristics. Performance measures were interpreted within clinically comparable task groups rather than ranked across the entire corpus. Accuracy, AUROC, AUPRC, F1-score, C-index, calibration, and regression-error measures were treated as distinct metrics.

A formal meta-analysis was not performed because of substantial clinical and methodological heterogeneity. Instead, a structured narrative synthesis was conducted in accordance with SWiM principles.

## 3 Results

The final synthesis comprised 169 unique eligible primary studies. Because individual studies frequently evaluated multiple algorithms, performance measures, feature-selection strategies, validation procedures, or explainability techniques, several characteristics were coded as multi-label variables and therefore do not necessarily sum to 100%. The results are presented according to the five research questions, beginning with study selection and overall characteristics of the evidence base, followed by analyses of modeling approaches and temporal prediction tasks, performance and validation, datasets and experimental settings, explainability, and methodological quality and clinical translation.

### 3.1 Study selection and characteristics of the evidence base

#### 3.1.1 Study selection

The literature search conducted in the Scopus database identified a total of 465 records from 2021 to 2026. Following the removal of nine duplicate records, 456 unique records remained and were screened based on their titles and abstracts. Of these, 200 records were excluded at the title and abstract screening stage because they did not meet the predefined eligibility criteria. Consequently, 256 articles were retrieved and assessed in full text for eligibility.

Following full-text assessment, 87 articles were excluded for specific reasons. 25 articles were published outside the predefined review period of 2021–2026, 10 were published in languages other than English, 29 did not involve an artificial intelligence or machine-learning-based application, 12 were not relevant to chronic kidney disease, and 11 were review articles rather than eligible primary research studies.

After applying all eligibility criteria, 169 studies met the inclusion criteria and were retained for the final systematic review and narrative synthesis. The complete study-selection process, including record identification, duplicate removal, title and abstract screening, full-text eligibility assessment, reasons for exclusion, and final study inclusion, is presented in the PRISMA flow diagram in Figure 2.

**Figure 2:**
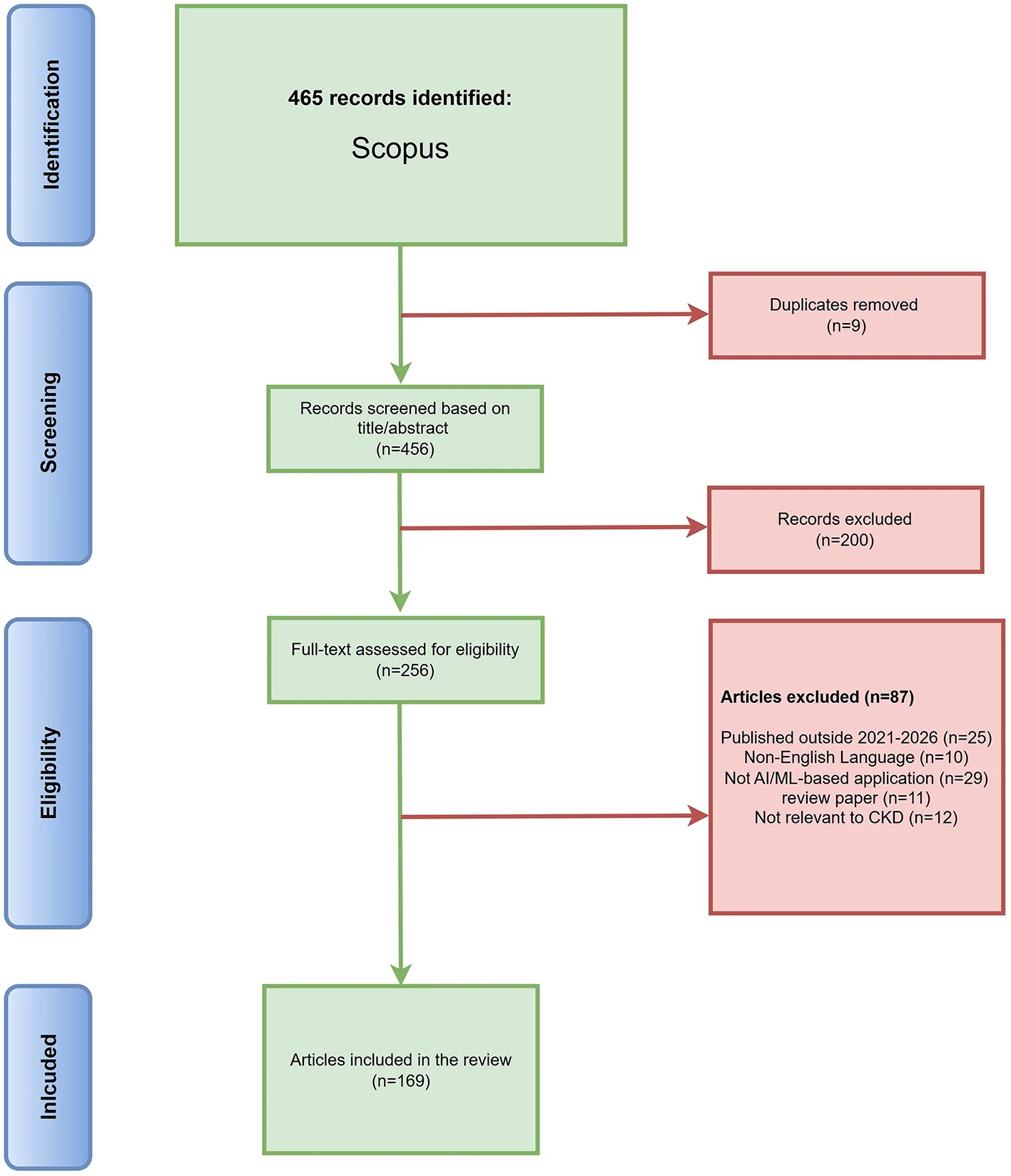
PRISMA-aligned flow of study identification, screening, eligibility assessment, and inclusion.

#### 3.1.2 Publication trends

The 169 included studies were published between 2021 and 2026. Publication activity increased substantially over the review period. Seven studies (4.1%) were published in 2021, 19 (11.2%) in 2022, 31 (18.3%) in 2023, 44 (26.0%) in 2024, 50 (29.6%) in 2025, and 18 (10.7%) in 2026. Thus, 112 studies (66.3%) were published between 2024 and 2026, indicating that approximately two-thirds of the evidence base emerged during the most recent 3 years covered by the review.

The 2026 total should be interpreted cautiously because it represents only the portion of the publication year captured by the search period and is therefore not directly comparable with complete preceding years (Table 5).

**Table 5:** Publication-year distribution of the 169 included studies.

Publication year	No. of studies	%
2021	7	4.1
2022	19	11.2
2023	31	18.3
2024	44	26.0
2025	50	29.6
2026	18	10.7
Total	169	100.0

#### 3.1.3 General characteristics of the included studies

The included literature was methodologically and clinically heterogeneous. Studies used public benchmark datasets, single-center and multicentre clinical cohorts, electronic health records, registries, population-based datasets, imaging, physiological signals, and molecular or biomarker data. The modeling approaches similarly ranged from conventional statistical and machine-learning algorithms to ensemble learning, deep neural networks, recurrent architectures, convolutional networks, and hybrid approaches.

The clinical objectives also differed substantially. Some studies evaluated contemporaneous CKD detection, diagnosis, or staging, whereas others investigated incident CKD, renal-function decline, disease progression, dialysis, kidney failure, or other future renal outcomes. This heterogeneity motivated the task-based synthesis used in the remainder of the Results rather than a single pooled comparison of model performance.

Accordingly, subsequent analyses were organized around the five research questions and explicitly considered prediction task, temporal design, dataset provenance, validation strategy, performance metric, explainability, and methodological quality.

### 3.2 RQ1: AI approaches and the temporal nature of CKD prediction

#### 3.2.1 Machine-learning and deep-learning model families

The included literature showed considerable diversity in model choice, but the distribution of algorithms was less informative than the context in which those algorithms were applied. Random forests, support-vector machines, logistic regression, boosting methods, decision trees, and neural-network architectures were repeatedly evaluated across the corpus, often within the same study as shown in Table 6. Consequently, model-family frequency primarily reflected the breadth of algorithm comparison rather than evidence that any one method was consistently superior.

**Table 6:** Major AI and machine-learning model families represented in the included studies.

Model family	No. of studies	References
Neural/deep-learning approaches	87	Mandava et al., 2024; Hassan et al., 2023; Bilal et al., 2024; Dey and Kamal, 2026; Nizamuddin et al., 2024; Usha and Kavitha, 2022; Iftikhar et al., 2026; El-Kenawy et al., 2024; Rubia et al., 2024; Ahmad et al., 2026; Venkatrao and·Shaik, 2024; Bhaskar et al., 2024; Jayaprabha and Vishwa Priya, 2024; Saif et al., 2024; Joteppa et al., 2024; Anoch and Parthiban, 2026; Liu et al., 2026; Yoshizaki et al., 2024; Bahrami et al., 2024; Yu et al., 2026; Su et al., 2022; Chowdhury et al., 2024; Takkavatakarn et al., 2023; Das·et al., 2026; Hoang et al., 2024; Jose and Sheeja, 2024; Nramban Kannan et al., 2024; Jai Kumar and Mohanasundaram, 2024; Wu Q. et al., 2025; Priyadharshini et al., 2025; Mamatha and Terdal, 2024; Suvaris et al., 2025; Boughougal et al., 2025; Brinda and Rajan, 2023; Leung et al., 2024; Suzuki et al., 2025; Tsai et al., 2025; Khafaga et al., 2025; Anbazhagan and Rangaswamy, 2025; Singamsetty et al., 2024; Stella and Vasanthi Kumari, 2024; Al-Majd et al., 2024; Li J. et al., 2025; Metherall et al., 2025; Qin et al., 2024; Xu et al., 2024; Hsu et al., 2025; Salakapuri et al., 2025; Khade et al., 2025b; Gopi et al., 2023; Jose and Fredrik, 2025; Lee et al., 2023; Binu and Devi, 2025; Chitra et al., 2023; Ohashi et al., 2025; Khade et al., 2023a; Bhak et al., 2025; Deepika et al., 2023; Wang et al., 2024; Islam et al., 2025; Ramesh et al., 2025; Pradeepa and Jeyakumar, 2023; Joo et al., 2023; Reddy et al., 2025; Pal, 2023b; Reddy et al., 2023; Zhu et al., 2023; Park et al., 2025; Majid et al., 2023; Aldossary, 2023; Schena et al., 2023; Fahim et al., 2025; Norouzi and Kahriman, 2024; Kwon et al., 2022; Hossain et al., 2021; Cao et al., 2022; Mohamed and Santhoshkumar, 2022; Zhang et al., 2021; Prayitnaningsih et al., 2026; Shu et al., 2026; Mohamed et al., 2022; Kanda et al., 2022; Elamparithi et al., 2025; Blankemeier et al., 2022; Lambert and·Perumal, 2022; Zhao et al., 2026; Khan et al., 2021
Random forest	65	Mandava et al., 2024; Hassan et al., 2023; Swathi et al., 2024; Yu et al., 2024; Liu et al., 2026; Bahrami et al., 2024; Phuong et al., 2026; Venkatarathinam et al., 2023; Su et al., 2022; Salau et al., 2023; Takkavatakarn et al., 2023; Bai et al., 2022; Colmenares-Mejía et al., 2024; Hoang et al., 2024; Liu et al., 2024; Jai Kumar and Mohanasundaram, 2024; Du et al., 2024; Afnaan, et al.; Baswaraj et al., 2024; Ajalkar et al., 2025; Okita et al., 2024; Singamsetty et al., 2024; Alturki et al., 2024; Saumya et al., 2025; Li J. et al., 2025; Mehta et al., 2024; Metherall et al., 2025; Xu et al., 2024; Müller-Kühnle et al., 2025; Bandyopadhyay et al., 2024; Tang et al., 2025; Imaduddin et al., 2026; Tsai et al., 2023; Gogoi and Valan, 2025a; Chitra et al., 2023; Anqui, 2025; Verma et al., 2025; Chen et al., 2025; Islam et al., 2025; Dhanwanth et al., 2023; Kanda et al., 2023; Pal, 2023b; Aldossary, 2023; Nateghi Haredasht et al., 2023; Surekha et al., 2023; Nishat et al., 2021; Seba and·Benifa, 2022; Li T. et al., 2025; Chaudhuri et al., 2021; Ilyas et al., 2021; Debal and Sitote, 2022; AlMohimeed, 2025; Cao et al., 2022; Elemam et al., 2025; Shu et al., 2026; Wu L.-h. et al., 2025; Voskergian et al., 2025; Prakash et al., 2022; Nneji et al., 2025; Zhou et al., 2025; Gogoi and Valan, 2025b; Gokiladevi et al., 2022; Kanda et al., 2022; Abdulrahman and Khlebis, 2025; Kwong et al., 2022
Boosting approaches	52	Bhaskar et al., 2024; Yu et al., 2024; Venkatarathinam et al., 2023; Ma et al., 2026; Yu et al., 2026; Su et al., 2022; Salau et al., 2023; Ghosh and Khandoker, 2024; Chuah et al., 2022; Takkavatakarn et al., 2023; Hoang et al., 2024; Koch Nogueira et al., 2023; Rani et al., 2024; Du et al., 2024; Boughougal et al., 2025; Ajalkar et al., 2025; Okita et al., 2024; Alturki et al., 2024; Saumya et al., 2025; Kusuma et al., 2025; Li J. et al., 2025; Yan et al., 2024; Xu et al., 2024; Müller-Kühnle et al., 2025; Bandyopadhyay et al., 2024; Tang et al., 2025; Imaduddin et al., 2026; Tsai et al., 2023; Gogoi and Valan, 2025a; Ganie et al., 2023; Verma et al., 2025; Raihan et al., 2023; Fu et al., 2023; Chen et al., 2025; Islam et al., 2025; Khade et al., 2023b; Dhanwanth et al., 2023; Zhao et al., 2025; Nateghi Haredasht et al., 2023; Seba and·Benifa, 2022; Li T. et al., 2025; Norouzi and Kahriman, 2024; Emmanuel et al., 2025; Debal and Sitote, 2022; Cao et al., 2022; Shu et al., 2026; Wu L.-h. et al., 2025; Voskergian et al., 2025; Nneji et al., 2025; Chiu et al., 2021; Gogoi and Valan, 2025b; Kanda et al., 2022
Support-vector machines	38	Mandava et al., 2024; Hassan et al., 2023; Dey and Kamal, 2026; Swathi et al., 2024; Kusumaningrum et al., 2024; Yu et al., 2024; Phuong et al., 2026; Su et al., 2022; Salau et al., 2023; Jai Kumar and Mohanasundaram, 2024; Afnaan, et al.; Baswaraj et al., 2024; Ajalkar et al., 2025; Yan et al., 2024; Xu et al., 2024; Salakapuri et al., 2025; Bandyopadhyay et al., 2024; Ghosh and Khandoker, 2023; Pal, 2023a; Wang et al., 2024; Islam et al., 2025; Pal, 2023b; Reddy et al., 2023; Majid et al., 2023; Aldossary, 2023; Surekha et al., 2023; Li T. et al., 2025; Norouzi and Kahriman, 2024; Chaudhuri et al., 2021; Debal and Sitote, 2022; AlMohimeed, 2025; Cao et al., 2022; Elemam et al., 2025; Wu L.-h. et al., 2025; Prakash et al., 2022; Nneji et al., 2025; Gokiladevi et al., 2022; Arumugam and Baghavathi Priya, 2022
Logistic regression	34	Mandava et al., 2024; Shi et al., 2022; Dey and Kamal, 2026; Yoshizaki et al., 2024; Ramírez Medina et al., 2024; Bai et al., 2022; Liu et al., 2024; Afnaan, et al.; Saumya et al., 2025; Yan et al., 2024; Gogoi and Valan, 2024; Bandyopadhyay et al., 2024; Tang et al., 2025; Imaduddin et al., 2026; Ghosh and Khandoker, 2023; Pal, 2023a; Chitra et al., 2023; Wang et al., 2024; Islam et al., 2025; Mishra et al., 2025; Dhanwanth et al., 2023; Nateghi Haredasht et al., 2023; Surekha et al., 2023; Zhu et al., 2025; Chaudhuri et al., 2021; Capitoli et al., 2025; Kumar et al., 2026; AlMohimeed, 2025; Elemam et al., 2025; Shu et al., 2026; Chiu et al., 2021; Gogoi and Valan, 2025b; Gokiladevi et al., 2022; Navarro-González et al., 2026
Decision-tree approaches	24	Swathi et al., 2024; Koch Nogueira et al., 2023; Afnaan, et al.; Baswaraj et al., 2024; Ajalkar et al., 2025; Imaduddin et al., 2026; Gogoi and Valan, 2025a; Pal, 2023a; Anqui, 2025; Islam et al., 2025; Reddy et al., 2023; Majid et al., 2023; Aldossary, 2023; Chaudhuri et al., 2021; Capitoli et al., 2025; Kumar et al., 2026; Ilyas et al., 2021; Debal and Sitote, 2022; AlMohimeed, 2025; Cao et al., 2022; Elemam et al., 2025; Chiu et al., 2021; Gogoi and Valan, 2025b; Gokiladevi et al., 2022
K-nearest neighbors	18	Swathi et al., 2024; Salau et al., 2023; Afnaan, et al.; Ajalkar et al., 2025; Saumya et al., 2025; Mehta et al., 2024; Salakapuri et al., 2025; Bandyopadhyay et al., 2024; Imaduddin et al., 2026; Wang et al., 2024; Islam et al., 2025; Reddy et al., 2023; Majid et al., 2023; Aldossary, 2023; Li T. et al., 2025; Kumar et al., 2026; Cao et al., 2022; Gokiladevi et al., 2022
Naive Bayes	16	Swathi et al., 2024; Bai et al., 2022; Das·et al., 2026; Jai Kumar and Mohanasundaram, 2024; Afnaan, et al.; Mehta et al., 2024; Salakapuri et al., 2025; Anqui, 2025; Wang et al., 2024; Pechprasarn et al., 2025; Majid et al., 2023; Aldossary, 2023; Bellocchio et al., 2021; Chaudhuri et al., 2021; AlMohimeed, 2025; Abdulrahman and Khlebis, 2025

Model-family categories are multi-label because individual studies often evaluated more than one algorithmic family.

A recurring pattern was that increasingly complex architectures were frequently applied to current-state CKD classification rather than to genuinely longitudinal prediction. For example, hybrid deep-learning, convolutional, ensemble, and optimization-based approaches were used to discriminate existing CKD from non-CKD participants (Mandava et al., 2024; Hassan et al., 2023; Bilal et al., 2024; Venkatrao and·Shaik, 2024; Bhaskar et al., 2024). Although these studies demonstrated the capacity of advanced models to capture non-linear patterns in structured CKD data, their clinical task remained primarily contemporaneous disease recognition.

In contrast, future-oriented studies did not depend on a single dominant algorithmic family. Incident CKD, progression, kidney failure, and dialysis were predicted using approaches ranging from logistic regression and random forests to boosting, recurrent neural networks, survival-oriented methods, and ensemble models. Yoshizaki et al. (2024) developed a machine-learning model for incident CKD using health-examination data, while some studies evaluated future end-stage kidney disease among patients with established CKD (Shi et al., 2022; Takkavatakarn et al., 2023; Bai et al., 2022). Hoang et al. (2024) similarly used a multicentre machine-learning framework to predict future maintenance-dialysis initiation. These studies indicate that the clinical relevance of a model depended more on temporal design and outcome definition than on algorithmic complexity alone.

Deep learning was most clearly justified when the input modality required automatic representation learning. Histopathology, electrocardiography, retinal imaging, chest radiography, and other image-based modalities were analyzed using deep architectures (Suzuki et al., 2025; Tsai et al., 2025; Hsu et al., 2025; Chen et al., 2026). This pattern suggests that deep learning contributed most meaningfully when it expanded the type of clinical information that could be modeled, rather than when it simply replaced classical classifiers on small tabular datasets.

#### 3.2.2 Clinical tasks and data modalities

The clinical tasks addressed by the included studies were highly heterogeneous and extended well-beyond a single concept of CKD “prediction.” A large component of the literature focused on prevalent CKD detection, diagnosis, or staging using contemporaneously measured laboratory and demographic variables. Representative examples include studies applying machine learning to clinical records, UCI-derived CKD data, and disease-classification datasets (Hassan et al., 2023; Swathi et al., 2024; Mehta et al., 2024). In these studies, the model primarily learned to reproduce a renal-status label that was already present at the time of predictor measurement.

A smaller but clinically distinct group of studies addressed outcomes that occurred after baseline assessment. Incident CKD was evaluated in health-examination and longitudinal diabetes populations (Yoshizaki et al., 2024; Chowdhury et al., 2024), whereas progression-oriented studies predicted future kidney-function decline, renal replacement therapy, ESKD, or dialysis (Inaguma et al., 2022; Takkavatakarn et al., 2023; Leung et al., 2024; Okita et al., 2024; Hoang et al., 2024). These studies differed fundamentally from contemporaneous classification because the prediction target was separated in time from the predictor measurements.

Data modality was closely related to the type of question being asked. Structured tabular data dominated benchmark classification and conventional clinical prediction, whereas longitudinal EHR data, imaging, ECG, histopathology, and biomarker-based data supported more specialized tasks. Zhu et al. (2023) used recurrent neural networks with electronic health records to model CKD progression, while Chen et al. (2026) used chest radiography to predict future moderate-to-severe kidney-function loss. Deep learning was also applied to retinal fundus images for CKD assessment (Hsu et al., 2025), to histopathological tissue characteristics (Suzuki et al., 2025), and to ECG signals for CKD-related risk modeling (Tsai et al., 2025).

Taken together, the evidence indicates that the CKD-AI literature cannot be interpreted as a single homogeneous prediction problem. Similar algorithms were applied to fundamentally different clinical targets, and similar performance metrics were reported for tasks ranging from current-state diagnosis to multi-year renal-outcome prediction.

#### 3.2.3 Temporal classification of CKD prediction tasks

The temporal classification revealed that the majority of studies described within the CKD “prediction” literature did not in fact forecast a future renal event. Of the 169 included studies, 122 (72.2%) were classified as T1 current-state detection or classification studies. These studies used predictors and CKD-related labels corresponding to approximately the same clinical time and therefore primarily addressed recognition of existing disease or renal status.

Only 17 studies (10.1%) were classified as T2 incident CKD prediction, in which predictor information preceded the subsequent onset of CKD. Twenty-four studies (14.2%) were classified as T3 progression or prognostic prediction, addressing later outcomes such as renal-function decline, ESKD, renal replacement therapy, or dialysis. Five studies (3.0%) combined contemporaneous assessment with a genuine future-oriented component and were classified as T4, while one study (0.6%) remained temporally ambiguous and was classified as T5.

The distinction between T1 and T2–T4 was evident at study level. Current-state studies used contemporaneous clinical measurements to classify CKD status (Hassan et al., 2023; Swathi et al., 2024; Mehta et al., 2024). In contrast, Yoshizaki et al. (2024) predicted incident CKD from baseline health-examination data; Chowdhury et al. (2024) evaluated future CKD risk in a retrospective longitudinal type 1 diabetes cohort; and also some studies modeled later progression to end-stage kidney disease (Shi et al., 2022; Takkavatakarn et al., 2023; Bai et al., 2022). Similarly, Hoang et al. (2024) predicted subsequent maintenance-dialysis initiation in a multicentre CKD cohort, while and other study used chest radiography to estimate future kidney-function loss (Chen et al., 2026).

For the broader synthesis, T1 studies were categorized as non-future-oriented (F0), T2–T4 studies as genuine future-oriented prediction (F1), and T5 as temporally ambiguous (FA). Accordingly, only 46 of the 169 studies (27.2%) contained a clearly genuine future-oriented prediction component. The remaining 122 studies (72.2%) primarily evaluated current renal status. This result indicates that the size of the published CKD “prediction” literature substantially exceeds the amount of evidence that actually predicts a subsequently occurring renal outcome. The distribution of the 169 included studies across the five temporal classes is summarized in Table 7.

**Table 7:** Temporal classification of the 169 included CKD-AI studies.

Class	Prediction task	*n*	References
T1	Current-state CKD detection/classification	122	Mandava et al., 2024; Hassan et al., 2023; Bilal et al., 2024; Dey and Kamal, 2026; Nizamuddin et al., 2024; Usha and Kavitha, 2022; Iftikhar et al., 2026; El-Kenawy et al., 2024; Rubia et al., 2024; Ahmad et al., 2026; Venkatrao and·Shaik, 2024; Bhaskar et al., 2024; Jayaprabha and Vishwa Priya, 2024; Swathi et al., 2024; Gupta et al., 2026; Joteppa et al., 2024; Anoch and Parthiban, 2026; Kusumaningrum et al., 2024; Liu et al., 2026; Bahrami et al., 2024; Sutariya and Rathod, 2024; Xue et al., 2026; Phuong et al., 2026; Venkatarathinam et al., 2023; Yadav and Sharma, 2023; Yu et al., 2026; Salau et al., 2023; Ghosh and Khandoker, 2024; Das·et al., 2026; Koch Nogueira et al., 2023; Jose and Sheeja, 2024; Nramban Kannan et al., 2024; Liu et al., 2024; Jai Kumar and Mohanasundaram, 2024; Rani et al., 2024; Priyadharshini et al., 2025; Mamatha and Terdal, 2024; Suvaris et al., 2025; Boughougal et al., 2025; Brinda and Rajan, 2023; Afnaan, et al.; Baswaraj et al., 2024; Ajalkar et al., 2025; Khafaga et al., 2025; Anbazhagan and Rangaswamy, 2025; Singamsetty et al., 2024; Stella and Vasanthi Kumari, 2024; Alturki et al., 2024; Saumya et al., 2025; Kusuma et al., 2025; Al-Majd et al., 2024; Mehta et al., 2024; Metherall et al., 2025; Qin et al., 2024; Müller-Kühnle et al., 2025; Hsu et al., 2025; Gogoi and Valan, 2024; Salakapuri et al., 2025; Bandyopadhyay et al., 2024; Khade et al., 2025a,b; Imaduddin et al., 2026; Ghosh and Khandoker, 2023; Gopi et al., 2023; Jose and Fredrik, 2025; Gogoi and Valan, 2025a; Lee et al., 2023; Binu and Devi, 2025; Pal, 2023a; Ganie et al., 2023; Anqui, 2025; Verma et al., 2025; Khade et al., 2023a; Raihan et al., 2023; Bhak et al., 2025; Deepika et al., 2023; Wang et al., 2024; Islam et al., 2025; Mishra et al., 2025; Ramesh et al., 2025; Khade et al., 2023b; Dhanwanth et al., 2023; Pradeepa and Jeyakumar, 2023; Reddy et al., 2025; Pechprasarn et al., 2025; Zhao et al., 2025; Pal, 2023b; Reddy et al., 2023; Park et al., 2025; Majid et al., 2023; Aldossary, 2023; Surekha et al., 2023; Nishat et al., 2021; Seba and·Benifa, 2022; Fahim et al., 2025; Zhu et al., 2025; Li T. et al., 2025; Norouzi and Kahriman, 2024; Chaudhuri et al., 2021; Bijoy et al., 2025; Hossain et al., 2021; Capitoli et al., 2025; Emmanuel et al., 2025; Kumar et al., 2026; Ilyas et al., 2021; Debal and Sitote, 2022; AlMohimeed, 2025; Mohamed and Santhoshkumar, 2022; Elemam et al., 2025; Prayitnaningsih et al., 2026; Wu L.-h. et al., 2025; Prakash et al., 2022; Nneji et al., 2025; Chiu et al., 2021; Mohamed et al., 2022; Gogoi and Valan, 2025b; Gokiladevi et al., 2022; Elamparithi et al., 2025; Abdulrahman and Khlebis, 2025; Lambert and·Perumal, 2022; Arumugam and Baghavathi Priya, 2022; Khan et al., 2021
T2	Incident CKD prediction	17	Saif et al., 2024; Yoshizaki et al., 2024; Ma et al., 2026; Su et al., 2022; Chowdhury et al., 2024; Colmenares-Mejía et al., 2024; Yan et al., 2024; Xu et al., 2024; Tang et al., 2025; Chitra et al., 2023; Joo et al., 2023; Nateghi Haredasht et al., 2023; Cao et al., 2022; Voskergian et al., 2025; Kanda et al., 2022; Blankemeier et al., 2022; Navarro-González et al., 2026
T3	CKD progression/prognosis	24	Shi et al., 2022; Yu et al., 2024; Ramírez Medina et al., 2024; Chuah et al., 2022; Takkavatakarn et al., 2023; Bai et al., 2022; Hoang et al., 2024; Chen et al., 2026; Du et al., 2024; Leung et al., 2024; Okita et al., 2024; Li J. et al., 2025; Tsai et al., 2023; Ohashi et al., 2025; Fu et al., 2023; Chen et al., 2025; Kanda et al., 2023; Zhu et al., 2023; Bellocchio et al., 2021; Schena et al., 2023; Shu et al., 2026; Zhou et al., 2025; Kwong et al., 2022; Zhao et al., 2026
T4	Mixed current-state and future-oriented prediction	5	Wu Q. et al., 2025; Suzuki et al., 2025; Tsai et al., 2025; Kwon et al., 2022; Zhang et al., 2021
T5	Temporally ambiguous longitudinal prediction	1	Inaguma et al., 2022
**Genuine future-oriented studies (T2–T4)**		**46**	Saif et al., 2024; Yoshizaki et al., 2024; Ma et al., 2026; Su et al., 2022; Chowdhury et al., 2024; Colmenares-Mejía et al., 2024; Yan et al., 2024; Xu et al., 2024; Tang et al., 2025; Chitra et al., 2023; Joo et al., 2023; Nateghi Haredasht et al., 2023; Cao et al., 2022; Voskergian et al., 2025; Kanda et al., 2022; Blankemeier et al., 2022; Navarro-González et al., 2026; Shi et al., 2022; Yu et al., 2024; Ramírez Medina et al., 2024; Chuah et al., 2022; Takkavatakarn et al., 2023; Bai et al., 2022; Hoang et al., 2024; Chen et al., 2026; Du et al., 2024; Leung et al., 2024; Okita et al., 2024; Li J. et al., 2025; Tsai et al., 2023; Ohashi et al., 2025; Fu et al., 2023; Chen et al., 2025; Kanda et al., 2023; Zhu et al., 2023; Bellocchio et al., 2021; Schena et al., 2023; Shu et al., 2026; Zhou et al., 2025; Kwong et al., 2022; Zhao et al., 2026; Wu Q. et al., 2025; Suzuki et al., 2025; Tsai et al., 2025; Kwon et al., 2022; Zhang et al., 2021

The study-level information on index time, predictor window, outcome timing, prediction horizon, and final temporal classification is provided in the Supplementary Table S2.

### 3.3 RQ2: performance, evaluation metrics, and validation

The reported performance of CKD-AI models was strongly conditioned by the clinical task, dataset source, and validation design. High discrimination was common in current-state classification, particularly on repeatedly used benchmark datasets, whereas future-oriented studies generally reported more moderate estimates under longitudinal or externally validated settings. This pattern suggests that raw performance values cannot be interpreted independently of the difficulty and independence of the evaluation setting.

#### 3.3.1 Reported performance by clinical task

Performance estimates differed systematically across prediction tasks. Current-state classification studies frequently reported very high accuracy or AUC, particularly when models were trained and tested on small benchmark datasets (Mandava et al., 2024; Hassan et al., 2023; Dey and Kamal, 2026; Ahmad et al., 2026; Kusumaningrum et al., 2024). These results indicate that established CKD labels can often be separated effectively when contemporaneous renal and laboratory variables are available, but they should not be interpreted as evidence of successful anticipation of future disease.

The performance profile changed when the outcome was temporally separated from the predictors. Incident CKD and progression studies generally reported lower, but clinically more informative, discrimination. Yoshizaki et al. (2024) reported strong short-term discrimination for incident CKD using baseline health-examination data, while some evaluated future CKD onset in longitudinal population cohorts (Su et al., 2022; Cao et al., 2022). Progression-oriented studies similarly reported moderate-to-high discrimination for ESKD, renal-function decline, or dialysis initiation rather than near-perfect separation (Takkavatakarn et al., 2023; Hoang et al., 2024; Leung et al., 2024; Li J. et al., 2025).

This contrast indicates that model performance tended to decrease as the prediction problem became more clinically demanding. The strongest numerical results were therefore not necessarily associated with the strongest predictive evidence. A model achieving almost perfect discrimination of an existing CKD label on a familiar benchmark dataset and a model achieving lower discrimination for kidney failure several years after baseline represent fundamentally different levels of predictive difficulty and clinical relevance.

#### 3.3.2 Evaluation metrics

Evaluation practices were dominated by discrimination and classification metrics. Accuracy was reported in 127 of the 169 studies, while AUC or AUROC appeared in 82. Recall or sensitivity was reported in 88 studies, precision in 68, and F1 or F-score in 68. By contrast, metrics more closely aligned with prognosis, probability calibration, or time-to-event prediction were much less common: C-index was reported in only four studies, Brier score in five, and AUPRC or PR-AUC in three.

The metric distribution mirrored the predominance of current-state classification. Accuracy, precision, recall, and F1-score are appropriate for categorical discrimination, but they provide limited information about whether predicted risks are numerically reliable or clinically useful. This limitation is particularly important for longitudinal CKD prediction, where a well-ranked patient may still receive a poorly calibrated absolute-risk estimate.

Future-oriented studies more frequently incorporated task-specific metrics. Time-dependent AUC or concordance measures were used in studies predicting CKD progression or incident disease (Yu et al., 2024; Ma et al., 2026), while longitudinal renal-function modeling sometimes used continuous-error measures such as RMSE or MAE (Tsai et al., 2023; Ohashi et al., 2025). Nevertheless, discrimination remained the dominant evaluation paradigm even in clinically oriented prediction studies.

The imbalance between discrimination and calibration therefore limits cross-study interpretation. Two models with similar AUROC values may differ substantially in estimated absolute risk, threshold behavior, and potential clinical consequences. Performance synthesis in this review was consequently interpreted according to the reported metric and prediction task rather than through direct numerical ranking across all studies.

#### 3.3.3 Validation strategies

Validation design provided a clearer indication of evidential strength than the headline performance value alone. Of the 169 studies, 136 did not report a clearly identifiable independent holdout or external validation strategy under the predefined coding rules. Twelve studies used an internal held-out split from the same underlying cohort, 17 reported geographic or institutional external validation, and only four were classified as using an independent external cohort or dataset. No study was coded as performing a distinct temporal validation using a later calendar period.

This distribution shows that most reported performance estimates were derived from development data or closely related samples. External validation was considerably more common among clinically oriented longitudinal studies than among benchmark classifiers. For example, external or institutional validation was reported for ESKD prediction (Takkavatakarn et al., 2023), dialysis initiation (Hoang et al., 2024), future kidney-function loss (Chen et al., 2026), CKD progression (Du et al., 2024; Li J. et al., 2025), ECG-based renal risk modeling (Tsai et al., 2025; Kwon et al., 2022), and retinal or imaging-based models (Hsu et al., 2025; Zhang et al., 2021).

The effect of validation was also visible in the reported results. Chen et al. (2026) for example, reported a decline in C-index from 0.903 internally to 0.851 under external validation, while Li J. et al. (2025) reported a reduction in AUC from 0.93 internally to 0.85 externally. Tsai et al. (2025) similarly reported lower external than internal AUC for ECG-based CKD modeling. These reductions should not be regarded as model failure; rather, they illustrate the expected difficulty of transporting a model beyond the population on which it was developed.

The strongest validation evidence came from the small subset using genuinely independent cohorts or datasets. These included incident CKD modeling based on biochemical and population data (Ma et al., 2026), nationwide CKD progression prediction (Fu et al., 2023), retina-derived CKD risk stratification (Joo et al., 2023), and progression prediction across diverse populations with type 2 diabetes (Zhao et al., 2026). Their numerical performance was not uniformly higher than that of internally evaluated models, but their estimates provide stronger evidence of transportability.

#### 3.3.4 Near-perfect performance and benchmark context

Near-perfect performance was concentrated mainly in current-state classification and benchmark-dependent studies. Several studies reported accuracy values approaching or reaching 100% (Mandava et al., 2024; Hassan et al., 2023; Dey and Kamal, 2026; Venkatrao and·Shaik, 2024; Kusumaningrum et al., 2024). Such results demonstrate that the benchmark labels can be highly separable under particular experimental pipelines, but they do not by themselves establish that the corresponding models will generalize to heterogeneous clinical populations.

The context of these results is important because many benchmark studies used overlapping versions of the same underlying CKD dataset and repeatedly optimized combinations of preprocessing, feature selection, resampling, and classifier selection. Therefore, multiple publications reporting near-perfect accuracy do not represent multiple independent confirmations of equivalent clinical performance. Instead, they often constitute repeated methodological experiments on the same patient-level evidence base.

This interpretation was supported by the contrast with externally evaluated clinical studies. Models tested across institutions, populations, or independent cohorts generally produced lower and more variable discrimination (Chen et al., 2026; Li J. et al., 2025; Tsai et al., 2025; Navarro-González et al., 2026). The difference suggests that the extremely high benchmark estimates were associated with easier and less independent evaluation settings rather than establishing superior clinical predictability.

Accordingly, the present review does not interpret near-perfect performance as evidence of bias or inflation in the absence of direct statistical proof. Instead, such estimates are treated as benchmark-specific results whose transportability remains uncertain until replicated on independent clinical populations.

#### 3.3.5 Dataset dependence and independence of evidence

The dataset-dependence analysis further changed the interpretation of the apparent volume of performance evidence. Sixty-seven studies were classified as reusing a public benchmark dataset, while one additional instance involved reuse of the same underlying clinical cohort across separate publications. Thus, a substantial fraction of the reported model evaluations were not based on independent patient populations.

This dependence was especially concentrated in current-state classification, where the same public CKD benchmark was repeatedly used to compare random forests, SVMs, neural networks, boosting algorithms, hybrid models, and optimization-based classifiers (Mandava et al., 2024; Hassan et al., 2023; Swathi et al., 2024; Gogoi and Valan, 2025a). Although these papers provide legitimate methodological comparisons, repeated success on the same underlying records should not be interpreted as equivalent to replication across different healthcare systems or populations.

By contrast, longitudinal clinical studies drew on a wider range of independent sources, including health-screening populations, multicentre cohorts, national databases, EHR networks, imaging cohorts, and population studies (Yoshizaki et al., 2024; Chowdhury et al., 2024; Hoang et al., 2024; Ma et al., 2026; Zhao et al., 2026). Their results were more heterogeneous, but they provide stronger evidence about performance under variations in patient characteristics, clinical setting, and outcome prevalence.

Taken together, the RQ2 findings show that the apparent superiority of CKD-AI models cannot be established from headline accuracy or AUROC alone. Performance was systematically intertwined with task difficulty, dataset reuse, validation independence, and metric choice. The most convincing evidence therefore came not from the highest reported numerical values, but from studies in which the prediction target was temporally meaningful and performance was evaluated on data sufficiently independent from model development.

### 3.4 RQ3: datasets, predictors, preprocessing, and experimental settings

The evidence base showed that data design was closely linked to the type of CKD problem being modeled. Public benchmark datasets supported much of the current-state classification literature, whereas genuine future-oriented prediction was concentrated in longitudinal clinical cohorts, registries, population studies, imaging cohorts, and other data sources in which predictors could be separated temporally from later renal outcomes. This distinction is important because the same modeling technique can represent either a relatively simple diagnostic classification problem or a clinically meaningful prognostic task depending on how the data are constructed.

#### 3.4.1 Dataset provenance

Dataset provenance showed a strong relationship with prediction temporality. Sixty studies used the classic UCI CKD benchmark or a direct mirror of its 400-case dataset, while a further 10 used other public benchmark datasets. Importantly, all 70 of these benchmark-based studies were classified as T1 current-state detection or classification; none constituted genuine future-oriented prediction. In contrast, future-oriented studies were drawn from longitudinal clinical cohorts, multicentre datasets, registries or EHR networks, population cohorts, imaging cohorts, and molecular or omics datasets as highlighted in Table 8.

**Table 8:** Dataset provenance across the 169 included CKD-AI studies.

Dataset provenance	*n*	References
UCI CKD benchmark/direct mirror	60	Mandava et al., 2024; Hassan et al., 2023; Bilal et al., 2024; Dey and Kamal, 2026; Usha and Kavitha, 2022; Rubia et al., 2024; Ahmad et al., 2026; Jayaprabha and Vishwa Priya, 2024; Swathi et al., 2024; Gupta et al., 2026; Joteppa et al., 2024; Sutariya and Rathod, 2024; Phuong et al., 2026; Venkatarathinam et al., 2023; Yadav and Sharma, 2023; Das·et al., 2026; Nramban Kannan et al., 2024; Jai Kumar and Mohanasundaram, 2024; Rani et al., 2024; Suvaris et al., 2025; Boughougal et al., 2025; Brinda and Rajan, 2023; Khafaga et al., 2025; Singamsetty et al., 2024; Kusuma et al., 2025; Al-Majd et al., 2024; Metherall et al., 2025; Gogoi and Valan, 2024; Bandyopadhyay et al., 2024; Khade et al., 2025a; Gopi et al., 2023; Gogoi and Valan, 2025a; Pal, 2023a; Verma et al., 2025; Raihan et al., 2023; Ramesh et al., 2025; Dhanwanth et al., 2023; Pradeepa and Jeyakumar, 2023; Pal, 2023b; Reddy et al., 2023; Majid et al., 2023; Aldossary, 2023; Surekha et al., 2023; Nishat et al., 2021; Seba and·Benifa, 2022; Chaudhuri et al., 2021; Kumar et al., 2026; Ilyas et al., 2021; AlMohimeed, 2025; Elemam et al., 2025; Prakash et al., 2022; Nneji et al., 2025; Mohamed et al., 2022; Gogoi and Valan, 2025b; Gokiladevi et al., 2022; Elamparithi et al., 2025; Abdulrahman and Khlebis, 2025; Lambert and·Perumal, 2022; Arumugam and Baghavathi Priya, 2022; Khan et al., 2021
Other Kaggle/public benchmark datasets	10	El-Kenawy et al., 2024; Kusumaningrum et al., 2024; Salau et al., 2023; Jose and Sheeja, 2024; Priyadharshini et al., 2025; Ajalkar et al., 2025; Imaduddin et al., 2026; Binu and Devi, 2025; Anqui, 2025; Fahim et al., 2025
Single-center clinical cohorts	55	Shi et al., 2022; Nizamuddin et al., 2024; Iftikhar et al., 2026; Venkatrao and·Shaik, 2024; Bhaskar et al., 2024; Yu et al., 2024; Liu et al., 2026; Xue et al., 2026; Yu et al., 2026; Ghosh and Khandoker, 2024; Chuah et al., 2022; Bai et al., 2022; Colmenares-Mejía et al., 2024; Hoang et al., 2024; Chen et al., 2026; Koch Nogueira et al., 2023; Liu et al., 2024; Mamatha and Terdal, 2024; Du et al., 2024; Baswaraj et al., 2024; Okita et al., 2024; Anbazhagan and Rangaswamy, 2025; Stella and Vasanthi Kumari, 2024; Alturki et al., 2024; Saumya et al., 2025; Yan et al., 2024; Mehta et al., 2024; Xu et al., 2024; Müller-Kühnle et al., 2025; Salakapuri et al., 2025; Khade et al., 2025b; Ghosh and Khandoker, 2023; Tsai et al., 2023; Jose and Fredrik, 2025; Ganie et al., 2023; Khade et al., 2023a; Deepika et al., 2023; Wang et al., 2024; Islam et al., 2025; Mishra et al., 2025; Khade et al., 2023b; Kanda et al., 2023; Reddy et al., 2025; Pechprasarn et al., 2025; Bellocchio et al., 2021; Nateghi Haredasht et al., 2023; Schena et al., 2023; Norouzi and Kahriman, 2024; Emmanuel et al., 2025; Debal and Sitote, 2022; Mohamed and Santhoshkumar, 2022; Shu et al., 2026; Chiu et al., 2021; Blankemeier et al., 2022; Kwong et al., 2022
Multicentre clinical cohorts	5	Leung et al., 2024; Chen et al., 2025; Kwon et al., 2022; Capitoli et al., 2025; Wu L.-h. et al., 2025
National/regional registries, claims databases, or large EHR networks	12	Saif et al., 2024; Inaguma et al., 2022; Takkavatakarn et al., 2023; Li J. et al., 2025; Chitra et al., 2023; Ohashi et al., 2025; Fu et al., 2023; Zhu et al., 2023; Li T. et al., 2025; Bijoy et al., 2025; Kanda et al., 2022; Navarro-González et al., 2026
Population cohorts or surveys	10	Yoshizaki et al., 2024; Bahrami et al., 2024; Ma et al., 2026; Su et al., 2022; Chowdhury et al., 2024; Tang et al., 2025; Joo et al., 2023; Zhao et al., 2025; Cao et al., 2022; Zhao et al., 2026
Imaging datasets or imaging cohorts	14	Anoch and Parthiban, 2026; Wu Q. et al., 2025; Afnaan, et al.; Suzuki et al., 2025; Tsai et al., 2025; Qin et al., 2024; Hsu et al., 2025; Lee et al., 2023; Bhak et al., 2025; Park et al., 2025; Zhu et al., 2025; Hossain et al., 2021; Zhang et al., 2021; Prayitnaningsih et al., 2026
Omics or molecular datasets	2	Ramírez Medina et al., 2024; Zhou et al., 2025
Synthetic datasets	1	Voskergian et al., 2025

This contrast indicates that benchmark reuse was not merely a question of sample source but was associated with a fundamentally different research objective. Benchmark studies typically optimized discrimination of an existing CKD label, often through repeated comparisons of classifiers, resampling procedures, feature-selection methods, or optimization algorithms (Mandava et al., 2024; Hassan et al., 2023; Swathi et al., 2024; Gogoi and Valan, 2025a). By comparison, studies using health-screening cohorts, EHRs, registries, and multicentre clinical data were more likely to estimate incident CKD, renal-function decline, dialysis initiation, or disease progression (Yoshizaki et al., 2024; Chowdhury et al., 2024; Hoang et al., 2024; Leung et al., 2024; Zhu et al., 2023; Cao et al., 2022).

The dataset-dependence audit reinforced this pattern. Sixty-seven studies reused public benchmark datasets, meaning that multiple publications evaluated different algorithms on substantially the same underlying patient records. These studies remain distinct methodological investigations, but they should not be interpreted as 67 independent clinical replications. Only one additional case involved reuse of the same underlying clinical cohort across separate publications. Consequently, the apparent breadth of evidence for some high-performing CKD classifiers is substantially greater at the publication level than at the level of independent patient populations.

#### 3.4.2 Predictor categories

Predictor choice also reflected the underlying clinical task. Current-state classification studies predominantly relied on structured laboratory and demographic variables that were already closely related to CKD diagnosis, whereas longitudinal studies more often used baseline clinical profiles, repeated measurements, imaging-derived features, or multimodal information to estimate later outcomes.

Routine renal and metabolic variables remained central across the literature. Serum creatinine, estimated glomerular filtration rate, blood urea measures, albuminuria-related variables, hemoglobin, blood pressure, age, diabetes-related measures, and other routinely available clinical factors were repeatedly incorporated into CKD models (Yoshizaki et al., 2024; Chowdhury et al., 2024; Cao et al., 2022; Zhao et al., 2025). In longitudinal studies, however, these variables were clinically more informative when their measurement clearly preceded the predicted renal event. For example, health-examination data were used to estimate later CKD incidence (Yoshizaki et al., 2024), while longitudinal laboratory measurements were used to model future renal-function change (Tsai et al., 2023) and EHR histories were used to predict CKD progression (Zhu et al., 2023).

A second pattern was the expansion from conventional tabular predictors to higher-dimensional modalities. Retinal fundus images were used for CKD detection and incidence prediction (Zhang et al., 2021), ECG signals were used for renal-impairment detection and subsequent risk assessment (Kwon et al., 2022; Tsai et al., 2025), chest radiography was used to estimate future kidney-function loss (Chen et al., 2026), and histopathological information was used to characterize renal injury and progression (Suzuki et al., 2025). Molecular and metabolomic predictors were less common but were generally deployed in more prognostic settings, including biochemical–metabolomic risk modeling and progression-related biomarker analysis (Ma et al., 2026). Thus, the principal difference was not simply the number of predictors available, but whether the predictors represented information obtainable before the outcome of interest.

#### 3.4.3 Preprocessing and missing-data handling

Preprocessing was not a uniform technical step across the reviewed studies; rather, it formed part of the modeling design and therefore affected the validity of the reported results. Common procedures included normalization or scaling, categorical encoding, missing-value imputation, removal of incomplete variables or records, class rebalancing, and modality-specific processing for images and physiological signals.

The handling of missing clinical data varied particularly widely. Some studies explicitly compared alternative missing-data strategies, including deletion and imputation approaches (Swathi et al., 2024), whereas longitudinal EHR studies employed more structured handling of incomplete repeated measurements before model development (Zhu et al., 2023). The methodological consequence is important: a model evaluated after aggressive imputation or data cleaning is not directly comparable with one developed on complete cases or routinely collected clinical records, even when both report the same discrimination metric.

Preprocessing was also strongly modality dependent. Imaging studies required resizing, normalization, segmentation or region-of-interest processing, augmentation, and feature extraction before classification or prognosis (Suzuki et al., 2025; Hsu et al., 2025; Zhang et al., 2021). Longitudinal EHR studies required temporal alignment and transformation of repeated measurements (Zhu et al., 2023; Tsai et al., 2023). These differences reinforce that preprocessing should be considered part of the predictive pipeline rather than treated as a neutral preliminary step.

A recurring limitation was that the ordering of preprocessing relative to training and evaluation was not always sufficiently clear. This matters because operations such as imputation, scaling, oversampling, or variable selection performed before data splitting can allow information from the validation or test set to influence model development. Accordingly, the review treated insufficiently specified preprocessing pipelines as a methodological uncertainty rather than assuming that leakage-free implementation had occurred.

#### 3.4.4 Feature selection and dimensionality reduction

Feature selection was particularly prominent in benchmark-oriented CKD classification, where relatively small tabular datasets were repeatedly subjected to wrapper, filter, embedded, optimization-based, and hybrid selection methods. Examples included evolutionary and metaheuristic optimization, statistical feature ranking, recursive or ensemble selection, and combinations of feature selection with resampling and explainability (Mamatha and Terdal, 2024; Gogoi and Valan, 2025a; Nneji et al., 2025).

These approaches frequently reduced the number of variables while maintaining or improving classification performance, but their interpretation requires caution. On small benchmark datasets, feature selection and classifier tuning are often performed within the same limited sample, meaning that gains may reflect increasingly efficient exploitation of a familiar dataset rather than evidence of improved transportability. This is particularly relevant where multiple studies repeatedly optimize different feature subsets on the same UCI-derived records.

By contrast, feature reduction in larger clinical and longitudinal datasets more often served a different purpose: improving parsimony, reducing measurement burden, or identifying clinically interpretable predictors. Pechprasarn et al. (2025) for example, explicitly examined CKD prediction using a minimal set of diagnostic predictors, while population and longitudinal studies used routine variables to construct models that could potentially be reproduced in screening or follow-up settings (Yoshizaki et al., 2024; Cao et al., 2022). The evidence therefore suggests that feature selection should be interpreted according to its purpose: optimization of benchmark classification and development of clinically parsimonious prediction tools are not equivalent objectives.

#### 3.4.5 Potential data leakage

The risk of information leakage was most relevant where the distinction between predictor and outcome was weak or where the analytical pipeline was incompletely described. Four recurring mechanisms were considered: use of variables that directly or indirectly encoded the CKD outcome, inclusion of post-index information in nominally predictive models, preprocessing or feature selection before train–test separation, and repeated observations from the same individual appearing across development and evaluation sets.

The first issue was particularly important in current-state classification. Many T1 studies used renal laboratory variables that contribute directly to the clinical definition or staging of CKD while simultaneously treating CKD status as the prediction target. Such models can be useful for automated classification, but high discrimination in this setting should not be interpreted as evidence of early prediction because the predictors may already contain information closely aligned with the outcome definition. This differs from longitudinal studies in which baseline variables were explicitly separated from later CKD onset or progression (Yoshizaki et al., 2024; Chowdhury et al., 2024; Hoang et al., 2024).

A second concern involved pipeline-level leakage. Feature selection, normalization, imputation, synthetic oversampling, and hyperparameter optimization are valid procedures when estimated exclusively from the training data, but they can produce optimistic estimates when information from the full dataset is used before evaluation. Several benchmark studies reported complex combinations of resampling, feature selection, and optimization (Swathi et al., 2024; Gogoi and Valan, 2025a; Mamatha and Terdal, 2024), making the sequencing of these steps especially important.

The synthesis therefore does not imply that leakage occurred in every study using these procedures. Rather, it identifies whether the reported experimental design was sufficient to rule it out. Taken together, the RQ3 findings show that dataset independence, temporal ordering of predictors and outcomes, and leakage-resistant preprocessing were more consequential for the credibility of CKD prediction than the use of increasingly elaborate feature-engineering or optimization methods alone.

### 3.5 RQ4: explainability and clinical interpretability

Explainability was uncommon across the 169 included studies and, where present, was predominantly technical rather than clinically validated. Only 16 studies reported an explicit explainability or interpretability approach, while the remaining 153 did not report a dedicated XAI method. More importantly, the presence of SHAP, LIME, feature importance, or an interpretable model did not in itself establish that the resulting explanations were clinically meaningful, stable, or useful in decision-making.

#### 3.5.1 Technical explainability methods

The explainability literature was concentrated in a small subset of studies as depicted in Table 9. SHAP was the most visible *post-hoc* approach, either used alone or in combination with LIME, while other studies relied on feature importance, fuzzy or intrinsically interpretable modeling, nomograms, or related explanation strategies. Representative applications included SHAP-based interpretation of CKD classifiers (Raihan et al., 2023; Surekha et al., 2023; Nneji et al., 2025; Gogoi and Valan, 2025b), combined SHAP–LIME analysis (Ghosh and Khandoker, 2024; Singamsetty et al., 2024), and broader interpretable modeling approaches (Gupta et al., 2026; Ghosh and Khandoker, 2023; Capitoli et al., 2025; Shu et al., 2026; Wu L.-h. et al., 2025).

**Table 9:** Explainability and interpretability approaches reported across the 169 included studies.

Explainability approach	*n*	References
SHAP	5	Ma et al., 2026; Raihan et al., 2023; Surekha et al., 2023; Nneji et al., 2025; Gogoi and Valan, 2025b
SHAP + LIME	2	Ghosh and Khandoker, 2024; Singamsetty et al., 2024
Feature importance only	1	Emmanuel et al., 2025
Other interpretable/explainable approaches	8	Gupta et al., 2026; Ghosh and Khandoker, 2023; Islam et al., 2025; Capitoli et al., 2025; AlMohimeed, 2025; Shu et al., 2026; Wu L.-h. et al., 2025; Khan et al., 2021
No explicit XAI method	153	—

The analytical value of these methods varied with the underlying prediction task. In current-state classification, explanation techniques frequently identified variables already known to be strongly associated with CKD, such as creatinine, eGFR-related measures, hemoglobin, blood pressure, albuminuria-related variables, age, and diabetes-related factors. Such results may improve transparency, but they do not necessarily provide new clinical insight if the model is primarily rediscovering variables closely related to the disease definition itself. This distinction is particularly important in benchmark studies, where apparently plausible feature rankings may coexist with limited evidence of temporal validity or external transportability.

In future-oriented prediction, explainability had greater potential to support clinical interpretation because explanations could be attached to risk estimates for later outcomes rather than merely to recognition of existing disease. For example, interpretable or explanation-oriented approaches were used in incident CKD modeling (Ma et al., 2026), dialysis-risk prediction (Shu et al., 2026), kidney-fibrosis prediction (Wu L.-h. et al., 2025), and population-level CKD risk analysis (Islam et al., 2025). In these settings, the explanatory task is clinically more demanding because important predictors must be interpreted as contributors to future risk rather than markers of disease already present at the time of assessment.

However, most XAI studies remained focused on attribution rather than validation of the explanation itself. SHAP and LIME can indicate which variables influence a model output, but they do not demonstrate that the identified relationships are causal, temporally stable, or clinically actionable. Consequently, technical interpretability was treated in this review as a property of the modeling pipeline rather than as evidence of clinical trustworthiness.

#### 3.5.2 Clinically validated interpretability

A clear gap emerged between technical explainability and clinically validated interpretability. None of the studies that used SHAP, LIME, feature importance, or related XAI methods demonstrated a comprehensive clinician-centered validation of the explanations themselves. In other words, the review identified studies that explained model behavior, but not studies showing that clinicians consistently judged those explanations to be accurate, useful, understandable, and beneficial for CKD decision-making.

This distinction is important because clinician comparison and explanation validation are not equivalent. Two studies compared model performance with clinicians or expert judgement (Chuah et al., 2022; Bellocchio et al., 2021), but neither was coded as using an explicit XAI method. Conversely, studies employing SHAP, LIME, or interpretable modeling generally did not test whether the explanations changed clinician decisions, improved diagnostic or prognostic accuracy, reduced uncertainty, or produced measurable clinical benefit.

The absence of such validation limits claims that explainable CKD-AI models are clinically trustworthy. A technically interpretable model may reveal which variables drive its predictions, but clinical trust also depends on whether those explanations are robust across populations, concordant with domain knowledge, appropriately calibrated to risk, and useful at the point of care. These dimensions were rarely evaluated together.

The synthesis therefore suggests that the main limitation in CKD-AI interpretability is not the complete absence of explanation methods, but the lack of progression from model-level explanation to clinician-validated interpretation. Future studies should move beyond reporting feature rankings or SHAP plots and evaluate whether explanations are reproducible, clinically coherent, and capable of improving human decision-making under realistic CKD workflows (Table 9).

### 3.6 RQ5: methodological quality, generalizability, and evidence for clinical translation

The principal limitation of the CKD-AI evidence base was not the absence of technically high-performing models, but the weakness of the methodological evidence supporting their transportability and clinical use. Across the corpus, high reported discrimination frequently coexisted with substantial risk of bias, limited independent validation, sparse calibration assessment, and little direct evaluation of clinical utility. Consequently, methodological maturity did not consistently increase alongside model complexity or reported performance.

#### 3.6.1 PROBAST+AI assessment

Methodological concerns were widespread across the 169 included studies. For model development, 156 studies (92.3%) were at high risk of bias and 13 (7.7%) unclear; none were low risk. For evaluation, 164 (97.0%) were high risk and five (3.0%) unclear. The analysis domain was the main source of concern (138 high-risk at development; 159 at evaluation), driven by small datasets, insufficient separation between development and evaluation, incomplete reporting, and limited independent validation. Participant-related concerns were also substantial (143 high-risk in both assessments), reflecting convenience samples, single-center cohorts, and reused benchmarks. Outcome-related concerns were less uniform (123 high-risk, 39 low, seven unclear), largely distinguishing longitudinal renal outcomes from contemporaneous or predictor-encoded outcomes. Predictor-related risk was rarely high (one study), but uncertainty was pervasive (168 unclear) due to poor reporting of timing, availability, preprocessing, and missing data. Applicability concerns were similarly cautious: 72 high and 97 unclear, with none low as demonstrated in Table 10. Overall, near-perfect accuracy or high AUROC should not be treated as strong evidence when the underlying study remains at high risk of bias; the evidence hierarchy favored temporally valid prediction, separated development and evaluation data, independent validation, transparent predictors, and clinically meaningful outcomes, even when numerical performance was more modest.

**Table 10:** Overall PROBAST+AI judgements across the 169 included studies.

Assessment	Low	Unclear	High
Development risk of bias	0	13	156
Evaluation risk of bias	0	5	164
Development applicability concerns	0	97	72
Evaluation applicability concerns	0	97	72

#### 3.6.2 External validation and generalizability

Generalizability was substantially weaker than the reported internal performance. Of the 169 studies, 17 used geographic or institutional external validation and only four used an independent external cohort or dataset; 136 lacked a clearly identifiable independent holdout or external validation strategy, 12 used an internal holdout, and none performed distinct temporal validation as presented in Table 11.

**Table 11:** Validation strategies across the 169 included CKD-AI studies.

Validation strategy	*n*	References
Geographic/institutional external validation	17	Xue et al., 2026; Ghosh and Khandoker, 2024; Takkavatakarn et al., 2023; Hoang et al., 2024; Chen et al., 2026; Liu et al., 2024; Wu Q. et al., 2025; Du et al., 2024; Tsai et al., 2025; Li J. et al., 2025; Hsu et al., 2025; Chen et al., 2025; Bellocchio et al., 2021; Zhu et al., 2025; Kwon et al., 2022; Zhang et al., 2021; Navarro-González et al., 2026
Independent external cohort/dataset validation	4	Ma et al., 2026; Fu et al., 2023; Joo et al., 2023; Zhao et al., 2026

External validation was concentrated in clinically oriented, future-focused studies, including prediction of ESKD, dialysis initiation, kidney-function decline, CKD progression, retinal or ECG-derived renal risk, and incident CKD (Takkavatakarn et al., 2023; Hoang et al., 2024; Chen et al., 2026; Wu Q. et al., 2025; Du et al., 2024; Tsai et al., 2025; Li J. et al., 2025; Chen et al., 2025; Joo et al., 2023; Bellocchio et al., 2021; Kwon et al., 2022; Zhang et al., 2021; Navarro-González et al., 2026; Zhao et al., 2026). Several studies showed lower performance after external testing (Chen et al., 2026; Li J. et al., 2025; Tsai et al., 2025), illustrating the greater difficulty of transferring models beyond the development setting.

The strongest evidence came from the four studies using independent external cohorts (Ma et al., 2026; Fu et al., 2023; Joo et al., 2023; Zhao et al., 2026). Overall, the findings show that high internal discrimination was common, but robust evidence of transportability across populations and settings remained limited. External validation was therefore considered methodologically stronger evidence even when its reported performance was lower than that of internally evaluated models.

#### 3.6.3 Calibration

Calibration was rarely assessed across the 169 included studies. Only 12 studies explicitly evaluated calibration, while 157 did not. This is important because strong discrimination does not necessarily mean that predicted risks correspond accurately to observed outcomes as summarized in Table 12.

**Table 12:** Studies reporting calibration assessment among the 169 included CKD-AI studies.

Calibration assessment	*n*	References
Calibration assessed	12	Ma et al., 2026; Hoang et al., 2024; Chen et al., 2026; Xu et al., 2024; Fu et al., 2023; Chen et al., 2025; Bellocchio et al., 2021; Li T. et al., 2025; Shu et al., 2026; Zhou et al., 2025; Navarro-González et al., 2026; Zhao et al., 2026
Calibration not reported	157	—

Calibration was concentrated mainly in clinically oriented and future-oriented studies, including models for incident CKD, dialysis initiation, future renal-function decline, post-nephrectomy CKD, diabetic CKD progression, and other prognostic outcomes (Ma et al., 2026; Hoang et al., 2024; Chen et al., 2026; Xu et al., 2024; Fu et al., 2023; Chen et al., 2025; Bellocchio et al., 2021; Li T. et al., 2025; Shu et al., 2026; Zhou et al., 2025; Navarro-González et al., 2026; Zhao et al., 2026). Some studies used Brier scores (Xu et al., 2024; Li T. et al., 2025; Shu et al., 2026), whereas others reported calibration curves or related assessments alongside discrimination and external validation (Hoang et al., 2024; Chen et al., 2026; Fu et al., 2023; Chen et al., 2025; Bellocchio et al., 2021; Zhao et al., 2026).

Notably, calibration often appeared in studies with stronger validation designs, suggesting that it was treated as part of more mature predictive evaluation rather than as a routine reporting practice. Overall, most CKD-AI studies established how well models discriminated between outcomes, but provided much less evidence that the predicted probabilities were sufficiently reliable for individualized risk communication or clinical decision-making.

#### 3.6.4 Clinical utility

Evidence of clinical usefulness was limited. Only 18 of the 169 studies moved beyond conventional model-performance reporting to some form of clinical comparison or use-oriented assessment. Six compared the proposed model with an established clinical score or baseline (Ramírez Medina et al., 2024; Hoang et al., 2024; Leung et al., 2024; Ohashi et al., 2025; Chen et al., 2025; Zhu et al., 2023), two compared model predictions with clinicians or experts (Chuah et al., 2022; Bellocchio et al., 2021), and only one explicitly evaluated clinical utility using decision-curve or net-benefit analysis (Li T. et al., 2025). A further nine demonstrated a workflow, application, dashboard, or related clinical-use interface.

This pattern shows that most studies established statistical discrimination rather than clinical benefit. In particular, evidence that model predictions could improve decision-making, referral, monitoring, or treatment allocation remained sparse.

#### 3.6.5 Deployment and translational evidence

Deployment evidence was similarly limited. Of the 169 studies, 159 reported no deployment evidence, while nine presented a prototype, system, application, or clinical-use tool (Joteppa et al., 2024; Gopi et al., 2023; Kanda et al., 2023; Aldossary, 2023; Li T. et al., 2025; Bijoy et al., 2025; AlMohimeed, 2025; Elemam et al., 2025; Navarro-González et al., 2026). Only one study was coded as demonstrating retrospective real-world operational use (Fu et al., 2023), and none reported a prospective clinical impact study.

Thus, the literature remains concentrated at the model-development and retrospective-validation stages. The existence of a web interface or prototype should therefore not be interpreted as evidence of clinical implementation or improved patient outcomes.

#### 3.6.6 Sensitivity analyses

Sensitivity analyses supported the distinction between the overall literature and the genuinely future-oriented evidence base. Among the 46 future-oriented studies, 16 underwent external validation and 11 assessed calibration, compared with 21 and 12 studies, respectively, in the full 169-study corpus. None of the future-oriented studies depended on the repeatedly reused public benchmark datasets identified in the dataset-dependence audit.

Restricting interpretation to future-oriented and more independent evidence therefore reduced the apparent dominance of benchmark classification studies and strengthened the relative contribution of longitudinal clinical cohorts. However, the overall conclusion remained unchanged: robust external validation, calibration, and direct evidence of clinical utility were still uncommon.

### 3.7 Summary of findings by research question

Across the five research questions, a consistent pattern emerged: the CKD-AI literature is methodologically diverse and often reports strong predictive performance, but the strength of evidence decreases when temporal validity, independent validation, calibration, and clinical translation are considered.

#### 3.7.1 RQ1—AI approaches and temporal nature

A wide range of conventional machine-learning and deep-learning models was identified, with neural/deep-learning, random-forest, and boosting approaches most frequently represented. However, model complexity was not the principal distinction in the literature. Of the 169 studies, 122 (72.2%) evaluated current-state CKD detection or classification, whereas only 46 (27.2%) contained a clearly future-oriented prediction component. Thus, much of the literature labeled as CKD “prediction” primarily addressed recognition of existing disease.

#### 3.7.2 RQ2—Performance, metrics, and validation

High accuracy and AUROC were frequently reported, particularly in benchmark-based current-state classification. However, performance was strongly dependent on task difficulty, dataset provenance, and validation design. Only 21 studies used geographic/institutional or independent external validation, while most relied on development data, internal resampling, or closely related samples. Consequently, lower externally validated performance often represented stronger evidence than near-perfect internal benchmark results.

#### 3.7.3 RQ3—Datasets and experimental settings

Dataset choice was closely associated with clinical task. Public benchmark datasets accounted for 70 studies and were concentrated entirely in current-state classification, whereas genuine future prediction relied predominantly on longitudinal cohorts, registries, EHRs, imaging, and population data. Reuse of common benchmark datasets also reduced the independence of the apparent evidence base. Predictor timing and the sequencing of preprocessing and feature selection were therefore more important to validity than increasingly elaborate feature-engineering pipelines alone.

#### 3.7.4 RQ4—Explainability and clinical interpretability

Explicit XAI was reported in only 16 studies and was dominated by SHAP, LIME, feature-importance, and related technical approaches. These methods improved transparency but rarely progressed to clinician-validated interpretation. The main gap was therefore not only limited use of XAI, but the absence of evidence that explanations improved clinical understanding or decision-making.

#### 3.7.5 RQ5—Methodological quality and clinical translation

PROBAST+AI identified high overall risk of bias in 156 studies during development and 164 during evaluation, with no study achieving an overall low-risk judgement. External validation, calibration, and clinical-utility assessment were uncommon, while prospective deployment evidence was essentially absent. Overall, the evidence base remains considerably stronger for model development and retrospective discrimination than for reliable clinical translation.

## 4 Discussion

This review shows that the rapid expansion of artificial intelligence for chronic kidney disease has produced a large and technically diverse literature, but the maturity of the evidence is considerably less uniform than the volume of publications or the magnitude of reported performance might suggest. Across 169 eligible studies, increasingly sophisticated machine-learning and deep-learning methods were frequently associated with high discrimination; however, the central methodological divide was not between classical and advanced algorithms. Rather, it was between studies that classified renal status already present at the time of assessment and those that established a temporally valid relationship between baseline predictors and a subsequently occurring renal outcome. This distinction changes the interpretation of what has often been described collectively as CKD “prediction.”

The temporal reclassification was particularly revealing. Nearly three quarters of the included studies were current-state detection or classification studies, whereas only 46 contained a clearly future-oriented component. Current-state studies can be valuable for automated detection, triage, or disease staging, but they should not be interpreted as evidence of early prediction when predictor measurements and CKD labels refer to essentially the same clinical time. This is especially important when renal variables closely related to the diagnostic definition of CKD are used to reproduce the disease label. By contrast, studies predicting incident CKD, ESKD, dialysis initiation, or future kidney-function decline from an identifiable baseline provide a stronger basis for claims of prognosis or early prediction (Yoshizaki et al., 2024; Takkavatakarn et al., 2023; Hoang et al., 2024; Chen et al., 2026; Zhao et al., 2026). The terminology used in future CKD-AI research should therefore reflect the actual temporal relationship between predictors and outcomes rather than using “prediction” as a generic synonym for classification.

This distinction also helps explain the apparent performance advantage of many benchmark-based studies. Near-perfect accuracy and AUROC were concentrated mainly in current-state classification using small, repeatedly analyzed public datasets (Mandava et al., 2024; Hassan et al., 2023; Dey and Kamal, 2026; Kusumaningrum et al., 2024). Such results demonstrate that these datasets can be highly separable under specific modeling pipelines, but they provide limited evidence about future clinical events or transportability to new populations. In contrast, longitudinal studies addressing more difficult outcomes generally reported less extreme performance, particularly when evaluated outside the development sample (Chen et al., 2026; Li J. et al., 2025; Tsai et al., 2025). The lower numerical performance of these studies should not be regarded as weaker evidence; in many cases, it reflects a more demanding and clinically meaningful prediction problem.

Dataset provenance further qualifies the apparent size of the evidence base. A substantial proportion of studies reused the same public benchmark datasets, meaning that numerous publications represent different methodological experiments on overlapping patient records rather than independent clinical replications. This does not diminish their value for algorithm development, but it limits the extent to which repeated high performance can be interpreted as independent confirmation. In contrast, future-oriented studies more often relied on population cohorts, EHRs, registries, multicentre clinical data, imaging cohorts, or other independently collected sources (Chowdhury et al., 2024; Leung et al., 2024; Zhu et al., 2023; Cao et al., 2022). Future reviews and primary studies should therefore distinguish publication-level replication from replication across genuinely independent patient populations.

The validation findings reinforce this concern. Only 21 studies used geographic, institutional, or independent external validation, and the strongest transportability evidence came from the very small subset evaluated on genuinely independent cohorts (Ma et al., 2026; Fu et al., 2023; Joo et al., 2023; Zhao et al., 2026). Several externally validated studies reported lower performance than during internal evaluation (Chen et al., 2026; Li J. et al., 2025; Tsai et al., 2025), illustrating the expected consequences of changes in case mix, measurement practice, prevalence, and clinical setting. Importantly, multicentre development should not automatically be interpreted as external validation when all participating sites contribute to training. The absence of distinct temporal validation also remains a major gap because model performance may deteriorate as clinical practice, population characteristics, and data-generation processes change over time.

Calibration and clinical utility were even less developed. Only 12 studies explicitly assessed calibration, despite the importance of reliable absolute-risk estimates for monitoring, referral, and treatment decisions (Ma et al., 2026; Hoang et al., 2024; Chen et al., 2026; Xu et al., 2024; Fu et al., 2023; Chen et al., 2025; Bellocchio et al., 2021; Li T. et al., 2025; Shu et al., 2026; Zhou et al., 2025; Navarro-González et al., 2026; Zhao et al., 2026). Similarly, very few studies moved beyond discrimination metrics to decision-curve analysis, comparison with established clinical tools, or clinician-centered assessment. Consequently, the literature provides considerably more evidence that models can separate outcomes statistically than that their risk estimates are sufficiently reliable or beneficial for clinical decision-making. Future studies should therefore treat calibration and decision-analytic evaluation as core components of prognostic model assessment rather than optional additions after AUROC or accuracy has been reported.

A similar gap was observed for explainability. Technical XAI methods such as SHAP, LIME, and feature-importance analysis were present in only a small subset of studies (Ma et al., 2026; Ghosh and Khandoker, 2024; Singamsetty et al., 2024; Raihan et al., 2023; Surekha et al., 2023; Nneji et al., 2025; Gogoi and Valan, 2025b), and their use rarely progressed to validation of whether explanations were meaningful or useful to clinicians. Feature attribution can increase transparency, but it does not establish causality, stability, calibration, or clinical benefit. This distinction is especially important when explanation methods highlight variables already closely related to the CKD definition. Clinical trustworthiness therefore requires more than visually plausible SHAP plots; explanations should be evaluated for robustness, clinical coherence, and their effect on human decision-making.

The risk-of-bias assessment provides a broader explanation for why strong reported performance has not yet translated into equally strong clinical evidence. PROBAST+AI identified high overall risk of bias in 156 studies during development and 164 during evaluation, with no study achieving an overall low-risk judgement. The analysis domain contributed particularly strongly to these assessments, reflecting concerns related to model development, evaluation, validation independence, and incomplete reporting of analytical procedures. These results should not be interpreted as implying that the models are technically ineffective. Rather, they indicate that the available evidence is generally insufficient to support confident estimates of how those models would perform when transferred to routine clinical practice.

Potential information leakage also deserves greater attention. The concern is not limited to overt use of future information; leakage may arise when outcome-related variables, preprocessing, imputation, feature selection, resampling, or hyperparameter optimisation are allowed to use information from data that subsequently serve as validation or test observations. Small benchmark studies combining several such steps are particularly vulnerable when the sequence of operations is incompletely described. Future CKD-AI studies should therefore report the full analytical pipeline, including when data splitting occurred and whether every preprocessing and feature-selection step was restricted to the training data.

Taken together, the evidence suggests that progress in CKD-AI should be judged less by increasingly complex architectures or marginal improvements in benchmark accuracy and more by improvements in study design. Priority should be given to clearly defined index times and prediction horizons, longitudinal outcomes, sufficiently large and representative cohorts, patient-level separation of development and evaluation data, external and temporal validation, calibration, comparison with established clinical tools, and assessment of clinical utility. Where explainability is used, its clinical interpretation should also be evaluated rather than assumed. These priorities would shift the field from demonstrating that AI can classify CKD-related patterns toward establishing whether it can provide reliable and actionable information before clinically important renal outcomes occur.

Overall, the literature demonstrates considerable technical capability but substantially less evidence of dependable clinical translation. The most important future advance is therefore unlikely to come from another marginally more complex classifier applied to a familiar benchmark dataset. Greater progress will come from temporally valid, independently evaluated, well-calibrated, and clinically assessed models that demonstrate not only whether CKD-related outcomes can be predicted, but whether those predictions remain reliable and useful in real-world care.

## 5 Limitations

The findings of this review should be interpreted in light of several methodological constraints. The review was restricted to the predefined Scopus-based search strategy and English-language studies, so relevant publications indexed elsewhere may have been missed. The heterogeneity of prediction tasks, datasets, outcomes, and metrics also precluded meaningful pooled comparison of model performance. In addition, incomplete reporting in primary studies sometimes limited confident assessment of predictor timing, preprocessing sequence, and validation design. The 169 included studies should therefore be interpreted as a comprehensive synthesis within the predefined review strategy rather than an exhaustive representation of every CKD-AI publication.

## 6 Future research directions

Future CKD-AI studies should move beyond contemporaneous disease classification and explicitly define an index time, predictor window, prediction horizon, and future renal outcome. Greater emphasis should be placed on incident CKD, progression, dialysis, kidney failure, and clinically meaningful renal decline rather than relabelling current-state detection as “prediction.”

Independent validation should become a routine requirement rather than an exception. Models should be tested across institutions, regions, and patient populations, with temporal validation using later calendar periods where possible. Multicentre development alone should not be considered sufficient evidence of transportability unless independent sites or cohorts are reserved for evaluation.

Future studies should evaluate not only discrimination but also whether predicted probabilities correspond to observed risks. Calibration plots, Brier scores, calibration intercepts or slopes, and decision-curve analysis should be incorporated more consistently. Clinical utility should also be assessed against established clinical scores, thresholds, referral strategies, or treatment decisions.

Public benchmark datasets remain useful for methodological development, but repeated optimization on the same small datasets provides limited evidence of clinical generalizability. Greater priority should be given to larger, longitudinal, multicentre, and independently collected clinical datasets, with clear reporting of dataset provenance and possible overlap between study populations.

Data splitting should occur before imputation, normalization, oversampling, feature selection, and hyperparameter optimization whenever appropriate. Patient-level separation should be ensured for repeated observations, and predictor availability should be verified relative to the intended prediction time. Complete reporting of the modeling pipeline would make leakage risk easier to assess and results easier to reproduce.

Future work should move beyond reporting SHAP values, LIME explanations, or feature rankings. Explanations should be evaluated for stability, clinical coherence, and usefulness to clinicians. Human-centered studies should assess whether interpretable outputs improve decision-making, reduce uncertainty, or support appropriate clinical action.

The field should progress from retrospective model development toward prospective evaluation in realistic clinical workflows. Studies should assess usability, workflow integration, decision impact, fairness, resource implications, and patient outcomes. Prospective deployment studies would provide stronger evidence of clinical translation than prototype interfaces or retrospective demonstrations alone.

Greater consistency is needed in reporting prediction tasks, datasets, temporal design, metrics, validation, calibration, and clinical utility. Future CKD-AI studies should align more closely with established prediction-model and AI reporting guidance so that models can be compared on the basis of methodological quality rather than headline accuracy alone.

## 7 Conclusion

This systematic review shows that the CKD-AI literature has advanced rapidly in model development, but the evidence supporting genuine early prediction and clinical translation remains comparatively limited. Across the 169 included studies, most investigations focused on current-state CKD detection or classification, while only a minority established a clear temporal relationship between baseline predictors and future renal outcomes. This distinction is critical because high performance in contemporaneous classification does not provide the same evidence as successful prediction of incident CKD, progression, dialysis, or kidney failure.

The review also demonstrates that headline accuracy or AUROC should not be interpreted in isolation. Reported performance was strongly shaped by prediction task, dataset provenance, benchmark reuse, validation design, calibration, and methodological quality. Many near-perfect results were obtained in small or repeatedly reused benchmark datasets, whereas independently validated and clinically oriented studies generally reported more modest but methodologically stronger performance. PROBAST+AI further showed that high risk of bias was widespread, while external validation, calibration, clinical-utility assessment, and prospective deployment were uncommon.

Taken together, the central challenge for CKD-AI is no longer simply to develop more complex models, but to generate more credible evidence. Future progress should prioritize temporally valid prediction tasks, independent and temporal validation, leakage-resistant analytical pipelines, reliable calibration, clinically meaningful evaluation, and prospective assessment in real-world settings. Explainability should likewise progress beyond technical attribution toward clinician-validated interpretation and decision support.

Overall, AI shows substantial potential to support CKD risk assessment and prognosis, but the current literature is considerably stronger for retrospective model development than for dependable clinical use. The field will move closer to meaningful clinical translation when success is judged not by increasingly high benchmark performance, but by whether models can provide accurate, transportable, well-calibrated, and clinically useful predictions before important renal outcomes occur.

## Data Availability

The original contributions presented in the study are included in the article/Supplementary material, further inquiries can be directed to the corresponding author/s.
